# Long-Term Warming of Baltic Sea Coastal Waters Affects Bacterial Communities in Bottom Water and Sediments Differently

**DOI:** 10.3389/fmicb.2022.873281

**Published:** 2022-06-10

**Authors:** Laura Seidel, Elias Broman, Magnus Ståhle, Emelie Nilsson, Stephanie Turner, Wouter Hendrycks, Varvara Sachpazidou, Anders Forsman, Samuel Hylander, Mark Dopson

**Affiliations:** ^1^Centre for Ecology and Evolution in Microbial Model Systems (EEMiS), Linnaeus University, Kalmar, Sweden; ^2^Department of Ecology, Environment and Plant Sciences, Stockholm University, Stockholm, Sweden

**Keywords:** climate change, marine waters, cyanobacteria, 16S rRNA gene amplicon sequencing, seasonal shifts

## Abstract

Coastal marine ecosystems are some of the most diverse natural habitats while being highly vulnerable in the face of climate change. The combination of anthropogenic influence from land and ongoing climate change will likely have severe effects on the environment, but the precise response remains uncertain. This study compared an unaffected “control” Baltic Sea bay to a “heated” bay that has undergone artificial warming from cooling water release from a nuclear power plant for ~50 years. This heated the water in a similar degree to IPCC SSP5-8.5 predictions by 2100 as natural systems to study temperature-related climate change effects. Bottom water and surface sediment bacterial communities and their biogeochemical processes were investigated to test how future coastal water warming alters microbial communities; shifts seasonal patterns, such as increased algae blooming; and influences nutrient and energy cycling, including elevated respiration rates. 16S rRNA gene amplicon sequencing and geochemical parameters demonstrated that heated bay bottom water bacterial communities were influenced by increased average temperatures across changing seasons, resulting in an overall Shannon's H diversity loss and shifts in relative abundances. In contrast, Shannon's diversity increased in the heated surface sediments. The results also suggested a trend toward smaller-sized microorganisms within the heated bay bottom waters, with a 30% increased relative abundance of small size picocyanobacteria in the summer (June). Furthermore, bacterial communities in the heated bay surface sediment displayed little seasonal variability but did show potential changes of long-term increased average temperature in the interplay with related effects on bottom waters. Finally, heated bay metabolic gene predictions from the 16S rRNA gene sequences suggested raised anaerobic processes closer to the sediment-water interface. In conclusion, climate change will likely alter microbial seasonality and diversity, leading to prolonged and increased algae blooming and elevated respiration rates within coastal waters.

## Introduction

Climate change is an ongoing process affecting all ecosystems on the planet that is accelerated as a result of increased anthropogenic greenhouse gas emissions, such as CO_2_ to the atmosphere (Sarmento et al., 2010). This leads to higher uptake of CO_2_ by global oceans with consequences, including acidification, increased salinity, stratification, sea-level rise, and temperature increase (Bindoff et al., 2019) that also reduces O_2_ solubility (Breitburg et al., 2018). Warmer oceans will also lead to higher metabolic rates of microorganisms, leading to higher O_2_ consumption (Brewer and Peltzer, 2017; Breitburg et al., 2018). How species diversity responds to global warming will differ based upon numerous variables, including the ecosystem, local versus global scales, and the composition of species (Rosset et al., 2010; Gruner et al., 2017). To date, the majority of experiment-based global climate change predictions have used small-scale, short-term, and fixed-temperature laboratory studies, which make it difficult to extrapolate the findings to predicting long-term, naturally fluctuating systems (Forsman et al., 2016).

Underlying knowledge of the responses of different ecosystems and their inhabiting organisms needs to be gathered to comprehend how global warming will affect the oceans. Hence, it is important to understand how microorganisms in sediment and water bodies will be affected, as they play crucial roles in energy and nutrient cycles by acting as both primary producers and decomposers (Field et al., 1998; Moran, 2015). Bacteria in bottom waters are found as either free living or particle associated, and their composition is highly influenced by environmental conditions, such as temperature, salinity, and resource availability (Fuhrman et al., 2008; Cui et al., 2019). In sediments, microbial communities are stratified due to the availability of electron acceptors (e.g., from O_2_ to ${\text{SO}}_{4}^{2-}$) for energy conservation that is influenced by, for example, temperature and/or oxygen availability (Nealson, 1997). Climate change effects combined with high-nutrient loads will also magnify the ongoing consequences of eutrophication by increasing seasonal phytoplankton blooms in duration and size (Reusch et al., 2018). These will be especially exacerbated within coastal areas, as shallow waters exchange CO_2_ more rapidly compared with deeper open oceans (Gilbert et al., 2010; Pavia et al., 2019). How the interactions between altered sediment communities and overlying bottom water and, e.g., the intensity, seasonality, and duration of algal and cyanobacterial blooms will be affected by global warming remains largely unknown.

A previous study by Seidel et al. (2022) studied the warm water discharge from a nuclear power plant that heated the bay for the last 50 years to average temperatures close to SSP5-8.5 (shared socioeconomic pathway) predictions of 3.3–5.7°C (IPCC, 2021). In the previous study, comparison of the heated bay to an unaffected control bay served as natural laboratories to study the effect of future climate change-related temperature increase on coastal sediment microbial communities in as close to natural conditions as possible. The study showed that prolonged warming of the microbial communities in the sediment in the heated bay exhibited shortened seasonal variation along with increased diversity due to a more rapid consumption of electron acceptors, which created shallowing of the redox tower for energy conservation *via* anaerobic electron acceptors and, consequently, a higher number of niches for anaerobic microorganisms (Seidel et al., 2022). The heated bay sediment communities also showed incomplete adaption to the prolonged warming by large numbers of stress and chaperone proteins, suggesting weakened resilience of the microbial communities (Seidel et al., 2022). These findings indicate a possible negative feedback loop with increased dead zones and accelerated sulfide generation closer to the sediment-water interface, leading to possible release of greenhouse gases in already existing oxygen-depleted zones. However, the previous study focused entirely on sediments and neglected effects on the pelagic zone.

In this study, we used the warm water discharge system to investigate and compare how bottom water samples collected between September 2017 and June 2018 were affected. Furthermore, we not only compared the two bays but looked closer at potential seasonal shifts. The posited questions were: (1) How does 50 years of warming influence microbial community diversity and abundances in the bottom water and surface sediment?; (2) does altered seasonality affect these microbial communities differently?; and (3) are nutrient and energy cycling potentially affected?

## Materials and Methods

### Model System and Sampling Sites

The model system used compares potential changes on microbial communities, which have undergone 50 years of increased and prolonged warming in the closest to natural possible conditions. For this, a nuclear power plant using Baltic Sea water for cooling and releasing the heated water into a semi-enclosed bay (only open to the open Baltic Sea) was chosen. The water from a nearby site is pumped through the system to cool down the reactor within a heat exchanger. During this process, the cooling water is not in direct contact with radioactive material and is during release, on average, 10°C warmer at the discharge site (Seidel et al., 2022). The heated water in the bay was, on average, 5.3–5.7°C warmer compared to the selected control bay with the largest differences in winter (December–February) and little to no difference in summer (June–September). The control bay was chosen based upon its proximity to the heated bay without being affected by the warmed water as it was not connected to the heated bay other than *via* ~1.5 km of open Baltic Sea. As described in Seidel et al. (2022), the two bays are natural systems, and environmental parameters may vary, such as a thin freshwater plume observed in the control bay surface waters during spring 2018. However, previous findings along with this study indicated temperature as a key driver in bacterial community selection.

**Figure 1:**
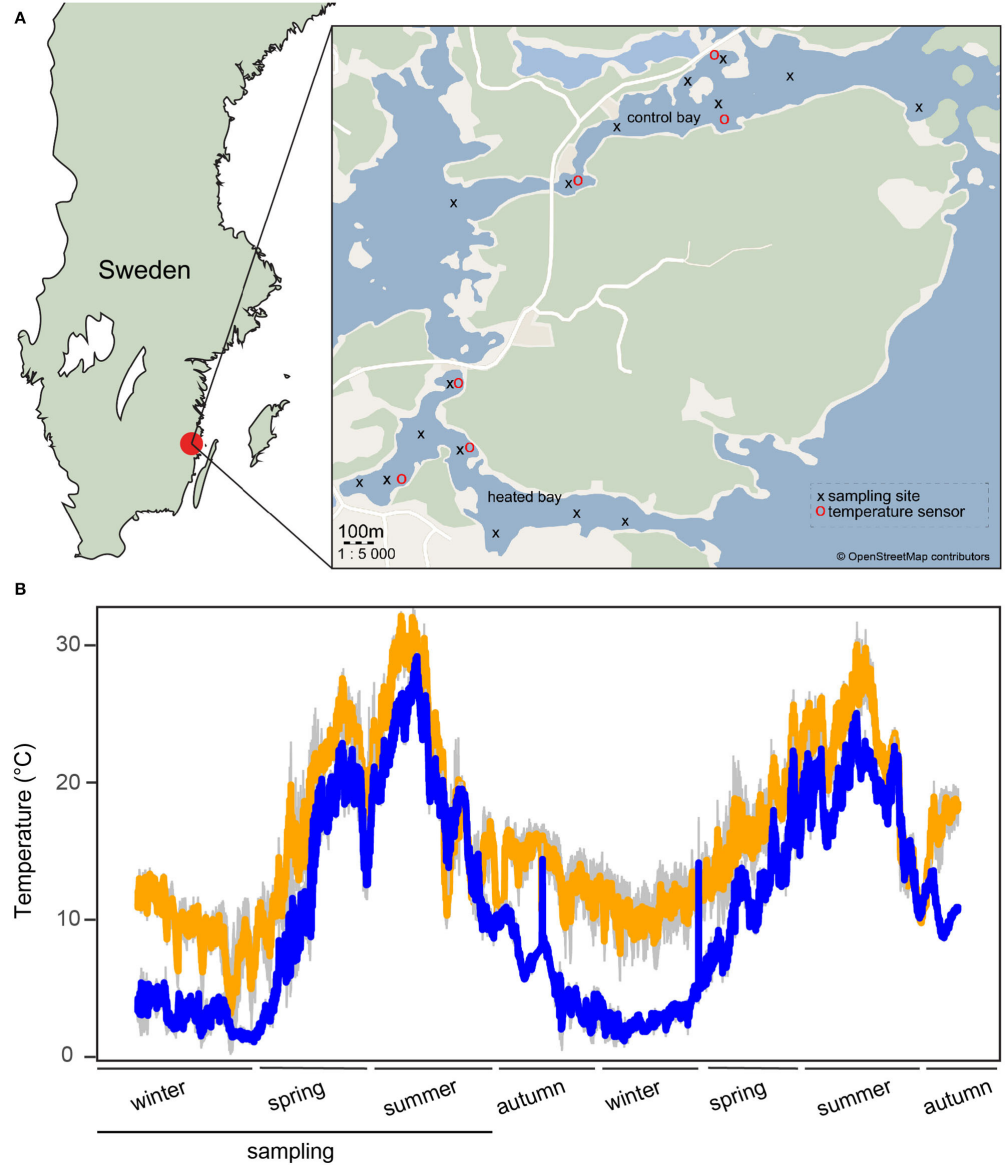
Sampling locations and temperature tracking. **(A)** A modified map from Seidel et al. (2022) of the sampling area in southern Sweden. Shown are the heated bay (south) and the control bay (north) with sampling sites marked with black crosses and temperature sensors with red circles. **(B)** A modified temperature graph from Seidel et al. (2022), showing the temperature variation at three different sampling sites per bay (1 m below the surface) measured from December 2017 until November 2019 at the heated bay (orange) and the control bay (blue) using HOBO data loggers. Standard deviations (both *n* = 3) are shown in gray. The lower black line shows the period of the present study from December 2017 to June 2018.

This study was composed of a time series of sampling in September, December, April, and June performed between September 2017 and June 2018 at the two coastal bays near the city of Oskarshamn, Sweden (temperature affected bay, 57°25'09.7“N 16°40'19.3”E and control bay 57°25'58.7“N 16°41'17.2”E; Supplementary Table 1). The samples did not overlap with those from the previous Seidel et al. (2022) study except for the six sediment samples taken in June 2018. Eight sampling sites per bay were selected with each site sampled in triplicate (Figure 1, Supplementary Table 1). The temperature was continuously measured at three sites per bay from December 2017 until November 2019 with data loggers (HOBOware, Onset Computer Corporation, USA). From the end of August 2017, warm water discharge was reduced for a 2-month period from 55 to 0.3 m3/sec. A description of data loggers set up, placement, and an overview of temperature changes between bays is given in Seidel et al. (2022) and Figure 1.

### Sediment and Bottom Water Sampling

Three replicate sediment cores were sampled with a Kajak gravity corer at each location to give 24 acrylic cores (inner diameter: 7 cm; length: 60 cm) for each bay and a total of 48 cores per sampling occasion as described in Seidel et al. (2022). Surface plus bottom water temperatures and bottom water oxygen concentrations were measured *in situ* (Multiline™ sensor, WTW™) during sampling. Additionally, overlying bottom water samples (BW; 50 ml) were taken from the sediment (SED) cores close to the SED surface and transferred into sterile tubes (Thermo Scientific™) for 16S rRNA gene amplicon sequencing (described below). An additional 15 ml of BW was filtered through a 0.7-μm Target2™ GMF Syringe Filter (Thermo Scientific™) into a pre-acid washed polypropylene tube (Thermo Scientific™) for chemical analyses. The BW was decanted from the core, and a 0–1-cm SED slice was collected in a sterile 50-ml polypropylene tube (Thermo Scientific™). The tube contents were well-mixed, and 15 ml was transferred to a pre-acid washed polypropylene tube (Thermo Scientific™) for subsequent pore water analysis. A further 2-ml reaction tube (Sarstedt, Inc.) was filled with SED for organic matter (OM) analysis. All samples were cooled during transport to the laboratory on the same day where they were frozen at either −80 or −20°C.

### Chemical Analysis

BW, pore water [wet sediment was centrifuged at 2,200 × g for 15 min and the water filtered through a 0.7- μM Target2™ GMF Syringe Filter (Thermo Scientific™)], and SED was analyzed. pH (pHenomenal, VWR pH electrode, VWR™) was measured for BW and pore water samples. Total Fe, ${\text{PO}}_{4}^{3-}$, and ${\text{SO}}_{4}^{2-}$ concentrations for pore water and BW were measured based on spectrophotometric methods (Broman et al., 2018). Additionally, ${\text{NO}}_{3}^{-}$ and ${\text{NO}}_{2}^{-}$concentrations for pore water were measured using Hach–Lange cuvette tests LCK339 and LCK341, respectively. Organic matter content (% wt) was measured using the loss on the ignition method as described in Broman et al. (2017b) except that the sample was dried before loss on ignition analysis for 6 days at 40°C instead of 3 days at 80°C.

### DNA Extraction, Sequencing, and Bioinformatics

A total of 50 ml of the BW samples was filtered through a 0.22-μM GV Durapore^®^ Membrane filter (Merck) and the filter retained for DNA extractions. BW and SED DNA extractions were performed using the DNeasy^®^ PowerWater and PowerSoil Extraction Kits (QIAGEN), respectively, according to the manufacturer's guidelines. DNA concentrations were measured using a Qubit^®^ 2.0 (Invitrogen™, Life Technologies Corporation). Amplifications of partial 16S rRNA gene fragments were conducted using the PCR primers 341f and 805r according to a modified PCR program by Hugerth et al. (2014), while library preparations were conducted according to Lindh et al. (2015) .

16S rRNA gene amplicon sequencing was performed at the Science for Life laboratory (SciLifeLab) in Stockholm, Sweden. Sequencing was done on the Illumina MiSeq platform with a 2 × 301 bp paired-end setup with an average of 213,555 raw sequences obtained (Supplementary Table 2). The raw data (with adapter removal and demultiplexing performed by the sequencing facility) were filtered, trimmed, denoised, merged, and chimeras removed with the DADA2 paired-end pipeline (v. 1.16) (Callahan et al., 2016) on the UPPMAX cluster (Uppsala Multidisciplinary Center for Advanced Computational Science). Trimming of the sequences was at 290 bp forward and 230 bp reverse, and left trimmed at 21 bp to ensure primer sequences were removed from the analysis. Maximum consistency of the error model was set to 30, and a minimum overlap at 10 bp with 0 mismatches was used for merging the forward and reverse reads. After removing chimeras using the default settings, the taxonomy was assigned against the Genome Taxonomy Data Base (GTDB; version 86) set for DADA2 (Parks et al., 2021). The average sequence output count was 75,180 reads (min., 366 and max., 289,850), with a total of 65,952 amplicon sequence variants (ASVs; Supplementary Table 2). The data were analyzed using R [version 4.0.4 (2021-02-15) (R Core Team, 2018)]. Packages used within the R environment are stated below at the specific steps they have been used. As the 16S rRNA gene sequencing data are compositional (Reimann et al., 2012) and follow Aitchison geometry due to the inherent closed structure of the data (Aitchison, 1986), the dataset was transformed to relative abundance per sample. A total of 37,916 unique ASVs in BW and 50,337 in SED were detected, of which 0.79% in BW and 1.77% in SED relative abundance were doubletons within the whole community, respectively. Alpha diversity analysis with removed doubletons resulted in a similar pattern and statistical results, and, therefore, the analysis was continued on the whole dataset (Supplementary Figure 1, **Table 3**).

Metabolic response prediction analysis with the PICRUSt2.0 tool (v. 2.4.0) (Douglas et al., 2020) was performed on counts based on significant (*p* < 0.05) differentially abundant ASVs from the abundance analysis described below. PICRUSt2.0 was used with default settings according to the tool guidelines available at: github.com/picrust/picrust2/wiki. There are certain key limitations to the prediction of functional profiles based on 16S rRNA gene amplicon sequences (github.com/picrust/picrust2/wiki/Key-Limitations), and, therefore, the results were limited to an overall comparison between bays. Analysis was performed on each dataset (BW and SED) separately to produce EC (enzyme commission number), KO (KEGG Orthology) identifiers, and pathway abundances per sample with KO identifiers used for further analyses.

### Statistical Analyses

Rarefaction curves were calculated with the vegan (Oksanen et al., 2019) (v. 2.5-6) package in R, suggesting that the majority of microbial diversity had been covered in both bays (Supplementary Figure 1). Furthermore, unknown Cyanobacterial sequences were run through additional annotation using the database Phytoref (2017-04-04), and sequences annotated as eukaryotic diatoms were removed. For further analysis, samples under 1,000 reads were removed, which included one sample in the BW (X2061; Supplementary Table 1) and two in SED sets (X51 and X11; Supplementary Table 1). For Alpha diversity analysis (Shannon's H, Shannon's H evenness, and Chao1 richness), the data were normalized by scaling with ranked subsampling (Beule and Karlovsky, 2020). To test the robustness of the analysis of Alpha diversity, the data were tested and visually compared without rarefying, rarefying the data to the smallest samples size (*n* = 2,356), as well as removing doubletons. All these tests resulted in similar patterns to the full dataset, showing strong validation of the results (Supplementary Figure 1). To test if the diversity indices were significantly different between the two bays over the different sampling months, ANOVA was performed. Due to the nature of the dataset, a linear mixed-effects model was used. Tested interactions showed months and bays were dependent upon each other and, therefore, were modeled as interaction and were treated as a fixed variable within the model. The different sampling sites, with replicates nested within each site, were treated as random variables. The variable sites was usually nested within bays as well but overfitted the model. The different models were tested and compared with the best fit chosen based on the Akaike information criterion (AIC). The model was created with the “lme()” function within the “nlme” package [v. 3.1-152; (Pinheiro et al., 2021)]. Pairwise comparison of the bays at each sampling month was performed using the “emmeans” package (v. 1.5.4) in R [Supplementary Table 3; (Lenth, 2021)] to test for significant differences between the bays at each sampling month.

Environmental variables of BW and SED (both *n* = 191) at the different sampling months between the different bays were shown within a radar plot from the ggplot2 package (v. 3.3.5) (Wickham, 2016) using the rescale function and the discrete scale option. A principal component analysis (PCA) of the environmental variables (*n* = 191 for both BW and SED) showed an association with season, bays, and sites for BW and SED (Supplementary Figure 2). This result indicated that the same model, which has been used for the diversity analysis, could also be used for testing statistical differences between the bays at the different sampling months within the environmental variables. Testing of the best fit using the AIC was performed and resulted in “bay” and “month” as fixed variables and modeled as interaction, while replicates were nested within a site as a random variable [“lme()” function, “nlme” package, v. 3.1-152, Supplementary Table 3]. For the higher resolution of the results of the environmental variables, boxplots, including all replicates and sites within each bay and each month (*n* = 24) plus the individual concentrations of each sample stated as points, were plotted with the ggplot2 package (Supplementary Figures 3, 4). To compare for significant differences between the surface and bottom water temperatures in each bay, a mixed-effects linear model and ANOVA were used. The temperature of surface and bottom waters was modeled with interaction to months and treated as fixed variables, while sampling site (*n* = 8) was treated as a random variable (Supplementary Table 3).

Statistical differences between both bays on bacterial communities were evaluated based on 999 permutations in the perMANOVA (permutational multivariate analysis of variance) based on the Bray-Curtis dissimilarities “adonis()” function within the “vegan” package (Oksanen et al., 2019; Supplementary Table 3). Within the perMANOVA, the variables bays and months were stated as interactions (bay × month), while the variable site was controlled by the “strata” option. Samples “X69,” “X78,” “X93,” “X1141,” “X1045,” “X2013,” “X2014,” and “X2015” (further details of the samples within Supplementary Table 1) were excluded from further distance-based redundancy analysis (db-RDA) of the bacterial communities with environmental variables as well as Pearson correlation. This was due to missing geochemical variables such that they did not increase the knowledge of interactions between the communities and environmental variables. Db-RDA (*n* = 187 BW, *n* = 185 SED) based on the relative abundance of ASVs and environmental parameters as explanatory variables was performed within R using the “vegan” package. ANOVA (*n* = 999 permutations) was used to test for statistically significant environmental variables. Results based on environmental variables (PCA in Supplementary Figure 2) and ordination plots of the microbial communities (Supplementary Figure 5) showed that seasons were the main driver of changes in bottom water communities, while surface sediment communities were less affected. Therefore, the permutations were performed within bays in BW to test for seasonal influence, while it was performed within months in SED to test for differences between bays. To test for microbial community dissimilarities between samples outside of a constrained ordination environment, the unconstrained ortholog to a db-RDA non-metric dimensional scaling (nMDS) was performed for BW and SED (Supplementary Figure 5).

SIMPER (similarity of percentages) analysis was performed to discriminate taxa (on an order level) responsible for the dissimilarities (based on Bray-Curtis dissimilarities) between the heated and control bay for BW and SED samples, using the “vegan” package (Supplementary Table 4).

Differential abundance analysis was performed on each dataset from the 16S rRNA gene ASVs within BW and SED samples (both *n* = 191). Taxa not identified in at least 50% of the total samples were filtered out (thus removing rare abundant ASVs), which resulted in *n* = 476 taxa retained for BW and *n* = 536 taxa for SED. A zero-inflated negative binominal model was used to account for a large number of zeros in the dataset. The analysis was carried out within the “DESeq2” package in R (version 1.30.1) based on counts to calculate possible statistical differences between ASVs of the two bays at the different sampling months (Supplementary Table 5; Love et al., 2014). To account for the nature of the dataset, the model used within the differential abundance analysis included bays, months, and sites as fixed factors (as DeSeq2 does not support random effects), while replicates were nested within sites. Significant (*p* < 0.05) differentially abundant ASVs over 1% from the previous step and environmental variables were used to calculate Pearson correlations (*n* = 191 BWand *n* = 163 SED). The counts were summarized on the order taxonomic level and clr (the centered log ratio) transformed using the packages “zComposition” (version 1.3.4) (Palarea-Albaladejo and Martín-Fernández, 2015) and “CoDaSeq” (version.99.6) (Gloor and Reid, 2016). Correlation was calculated using the “ggcormat” function from the “ggstatsplot” package (Patil, 2018) with Benjamin–Hochberg correction for *p*-values (Supplementary Table 6).

To test for potential differences between the two bays within bottom water and surface sediments, a differential abundance analysis on predicted abundances of the KO identifiers based on PICRUSt2.0 predictions was performed using the ALDEx2 (version 1.22.0) package in R (Gloor et al., 2016). The differential abundance analysis tool was used as it was suggested by the creator of the PICRUSt analysis tool. The analysis was performed for each dataset (BW and SED) on default settings with “bay” as condition setting. Only significant (*p* < 0.05) results from Welch's *t*-test and the Wilcoxon rank test were retained (Supplementary Table 8).

## Results

BW and surface SED (0–1 cm) sampling on four occasions between September 2017 and June 2018 within a “heated” (temperature affected), and a “control” bay generated 96 samples per bay from BW (*n* = 192) plus SED (*n* = 192) for geochemical analysis and 16S rRNA gene amplicon sequencing.

### Geochemical Parameters in the Two Bays

Both the bottom and surface water temperatures were significantly higher in the heated bay compared to the control bay [mixed linear model, ANOVA, *F*_(1, 14)_ = 331.8, *p* < 0.0001 surface water and *F*_(1, 14)_ = 227.7, *p* < 0.0001 BW; Supplementary Table 3]. This difference was, on average, 5.7 and 5.3°C higher in the BW and surface waters, respectively (Figure 1, Supplementary Table 1). Furthermore, temperature fluctuations in the control bay were greater due to warm water discharge, limiting heated bay winter lows. The water from the cooling outlet was, on average, 10°C warmer compared to the ambient water temperature except during the period of 18 August−22 October 2017 when the reactor was turned off and similar water temperatures were observed between the bays. However, microbial community and chemistry comparisons were not strongly influenced during this period (Supplementary Figures 2, 5), suggesting changes did not rapidly occur, and the reactor shutdown did not notably influence the data.

**Figure 2:**
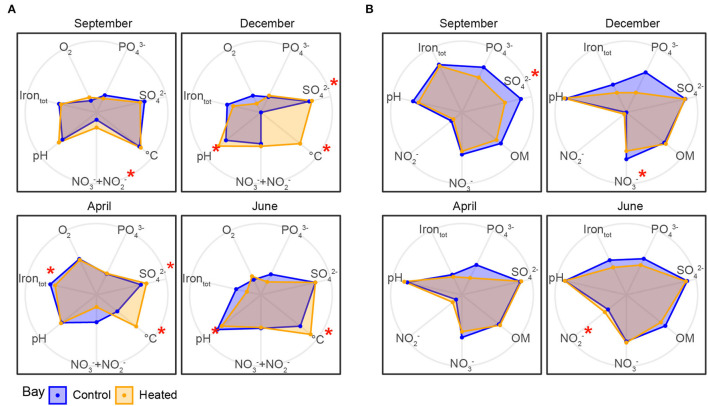
Geochemical parameters in the heated (orange) and control (blue) bays in bottom water and sediment pore water over the four different sampling occasions. Geochemical parameters were measured in the bottom water **(A)** and sediment pore water **(B)** in the two bays between September 2017 and June 2018 (total 24 pore water samples from 0 to 1 cm sediment and 24 bottom water samples per bay for each month, *n* = 192 for both bottom and pore waters). Radar plots were created with R using log_10_ scale grouped by bays. Significantly different environmental variables between the bays are marked with a red asterisk. Low values of the environmental variables are closer to the center of the radar plot, while higher concentrations are further toward the edges; values were rescaled using the “rescale” function of ggplot2.

Salinity concentrations were significantly higher in the surface [mean + s.d., a 6.6 ± 0.2% heated bay vs. a 4.8 ± 1.4% control bay, a linear mixed model, ANOVA, *F*_(1, 14)_ = 43.4, *p* < 0.0001] and bottom waters [6.6 ± 0.2% vs. 6.1 ± 0.6%, *F*_(1, 14)_ = 8.6, *p* = 0.01] of the heated bay compared to the control bay (Table 1, Supplementary Table 3). Oxygen concentrations were similar in the two bays [9.9 ± 5 vs. 10.9 ± 4.9 mg/L, *F*_(1, 14)_ = 0.1, *p* = 0.712]. Similarly, there was no significant difference in sediment organic matter (%) between the two bays (32 ± 13.3 vs. 34.6 ± 13.3%, *F*_(1, 14)_ = 0.1, *p* = 0.81; Figure 2, Table 2, Supplementary Figure 4, Supplementary Table 3].

**Table 1:** Salinity concentration and pH in surface and bottom waters.

		**Surface**		**Bottom**	
		**Heated bay**	**Control bay**	**Heated bay**	**Control bay**
		**Mean** **±SD (%)**			
September	Salinity	–	–	–	–
December		6.80 ± 0.05	5.31 ± 0.77	6.89 ± 0.22	6.36 ± 0.39
April		6.60 ± 0.09	3.24 ± 1.06	6.58 ± 0.10	5.86 ± 0.89
June		6.48 ± 0.18	5.75 ± 0.61	6.44 ± 0.07	6.11 ± 0.34
September	pH	–	–	8.33 ± 0.39	8.12 ± 0.34
December		7.87 ± 0.10	7.82 ± 0.27	8.60 ± 0.34	8.23 ± 0.27
April		8.52 ± 0.52	7.83 ± 0.17	8.19 ± 0.38	8.20 ± 0.28
June		8.54 ± 0.21	8.40 ± 0.03	8.37 ± 0.21	8.66 ± 0.17

*An overview of the average (mean ± sd) salinity concentrations in parts per thousand (%) and pH in surface and bottom waters of the heated (n = 8) and control (n = 8) bays. The data were collected in December, April, and June (September data are missing)*.

**Table 2:** Environmental variables.

**Bottom water**				**Sediment**			
**Bay**	**Month**	**Variable**	**Mean ±s.d**.	**Bay**	**Month**	**Variable**	**Mean ±s.d**.
Heated	September	Oxygen (mg/L)	8.75 ± 2.35	Heated	September	Nitrate (μM)	19.66 ± 13.38
	December		7.03 ± 1.29		December		20.58 ± 14.24
	April		16.83 ± 12.23		April		22.68 ± 22.92
	June		8.69 ± 1.04		June		39.60 ± 20.29
Control	September		7.80 ± 2.38	Control	September		25.80 ± 17.45
	December		9.30 ± 0.72		December		37.45 ± 14.85
	April		18.56 ± 3.62		April		31.94 ± 24.78
	June		8.69 ± 2.85		June		36.01 ± 21.03
Heated	September	Nitrate + nitrite (in combination) (μM)	2.02 ± 2.23	Heated	September	Nitrite (μM)	0.36 ± 0.34
	December		2.74 ± 1.18		December		0.10 ± 0.19
	April		0.97 ± 0.56		April		0.47 ± 0.40
	June		2.68 ± 1.80		June		1.91 ± 3.27
Control	September		0.67 ± 1.18	Control	September		0.51 ± 0.35
	December		2.51 ± 0.60		December		0.03 ± 0.08
	April		2.35 ± 1.35		April		0.37 ± 0.49
	June		2.69 ± 0.87		June		1.15 ± 0.40
Heated	September	Total iron (μM)	2.10 ± 0.72	Heated	September	Total iron (μM)	15.56 ± 4.58
	December		1.40 ± 0.33		December		3.38 ± 1.58
	April		2.86 ± 0.23		April		3.04 ± 0.66
	June		0.65 ± 0.53		June		5.37 ± 3.64
Control	September		2.38 ± 0.25	Control	September		17.19 ± 3.76
	December		1.97 ± 0.95		December		5.03 ± 2.45
	April		3.97 ± 1.32		April		3.28 ± 0.58
	June		1.18 ± 1.83		June		7.45 ± 4.69
Heated	September	Sulfate (mM)	2.22 ± 0.60	Heated	September	Sulfate (mM)	1.57 ± 0.19
	December		3.11 ± 0.80		December		3.70 ± 1.22
	April		2.75 ± 0.71		April		3.98 ± 0.67
	June		3.47 ± 0.57		June		3.90 ± 0.64
Control	September		2.55 ± 0.66	Control	September		3.97 ± 0.76
	December		2.68 ± 0.57		December		3.89 ± 1.30
	April		2.25 ± 0.41		April		3.87 ± 0.83
	June		3.43 ± 0.50		June		4.22 ± 0.51
Heated	September	Phosphate (μM)	10.62 ± 2.15	Heated	September	Phosphate (μM)	120.81 ± 81.10
	December		10.58 ± 2.07		December		64.71 ± 72.00
	April		12.22 ± 3.79		April		54.31 ± 65.53
	June		9.55 ± 1.34		June		92.45 ± 116.09
Control	September		10.98 ± 1.67	Control	September		205.19 ± 129.09
	December		10.30 ± 1.26		December		143.05 ± 102.65
	April		11.27 ± 2.44		April		95.68 ± 76.93
	June		11.34 ± 5.89		June		115.66 ± 98.74
Heated	September	Temperature (°C)	15.97 ± 0.96	Heated	September	OM (%)	29.80 ± 11.89
	December		12.92 ± 1.34		December		38.06 ± 12.86
	April		13.33 ± 0.77		April		39.93 ± 17.28
	June		19.91 ± 1.61		June		28.01 ± 13.81
Control	September		15.25 ± 0.56	Control	September		33.30 ± 11.70
	December		3.73 ± 1.24		December		33.30 ± 13.99
	April		7.13 ± 1.39		April		38.85 ± 16.32
	June		13.25 ± 2.08		June		35.77 ± 10.91

*An overview of the average (mean ± sd) environmental variables concentrations in bottom waters and sediments of the heated (n = 8) and control (n = 8) bays. The data were collected in September, December, April, and June*.

Bottom water ${\text{NO}}_{3}^{-}$/${\text{NO}}_{2}^{-}$ concentrations (Figure 2, Table 2) were significantly higher in the heated bay during September (a 2.02 ± 2.2-μM heated bay vs. a 67 ± 1.2-μM control bay, pairwise comparison, *p* = 0.005; Supplementary Table 3) and had significantly lower concentrations in April (0.97 ± 0.6 vs. 2.35 ± 1.3 μM, *p* = 0.044). In addition, significantly higher heated bay ${\text{SO}}_{4}^{2-}$ concentrations were measured during April (2.75 ± 0.7 vs. 2.3 ± 0.4 mM, *p* = 0.013) and December (3.1 ± 0.8 vs. 2.7 ± 0.6 mM, *p* = 0.028). Furthermore, significantly lower concentrations of heated-bay totally dissolved Fe were measured during April (2.9 ± 0.23 vs. 3.9 ± 1.3 μM, *p* = 0.0077; Figure 2, Table 2 plus Supplementary Figure 3, Supplementary Table 3). The ${\text{SO}}_{4}^{2-}$ concentrations in both bays ranged between 2 and 5 mM (Figure 2, Table 2, Supplementary Figure 3). BW ${\text{PO}}_{4}^{3-}$ was not significantly different between the bays [10.6 ± 2.5 vs. 11.1 ± 3.5 μM, ANOVA, *F*_(1, 14)_ = 0.21, *p* = 0.65].

A significant decrease in concentration was measured in heated-bay sediment of ${\text{NO}}_{3}^{-}$in December (20.5 ± 14.2 vs. 37.45 ± 14.8 μM, pairwise comparison, *p* = 0.0182), and ${\text{SO}}_{4}^{2-}$ concentrations [3.70 ± 1.2 vs. 3.89 ± 1.3 mM, ANOVA, *F*_(1, 14)_ = 23.3, *p* < 0.0001; Figure 2, plus Supplementary Figure 4, Supplementary Table 3]. The lower concentration of sediment ${\text{NO}}_{3}^{-}$ in the heated bay indicated increased microbial activity, resulting in lower (but not significantly) total dissolved Fe concentrations (3.38 ± 1.5 vs. 5.03 ± 2.4 μM, *p* = 0.1493) and increased use of ${\text{SO}}_{4}^{2-}$ as an alternative electron acceptor in December (Figure 2, Table 2, plus Supplementary Figure 4, Supplementary Table 3). Heated-bay sediment pore water ${\text{PO}}_{4}^{3-}$ was lower (but not significantly) compared to the control bay [a 63.1 ± 74.7 μM heated bay, a 125.9 ± 104.9 μM control bay, ANOVA, *F*_(1, 14)_ = 1.8, *p* = 0.21; Figure 2, Supplementary Table 3] during the whole year and showed the highest difference in September and December (Table 2, Supplementary Figure 4).

In summary, the heated bay bottom waters showed overall higher temperatures with less fluctuations over the sampling months. Additionally, higher salinity concentrations were observed within the heated bay bottom waters, with similar oxygen concentrations compared to the control bay, due to continuous mixing of the water column. Furthermore, the heated bay showed decreased ${\text{NO}}_{3}^{-}$and ${\text{SO}}_{4}^{2-}$ concentrations during winter (December), indicating the use of alternative electron acceptors plus lower ${\text{PO}}_{4}^{3-}$ concentrations compared to the control bay.

### Microbial Diversity in Sediment and Bottom Waters

**Figure 3:**
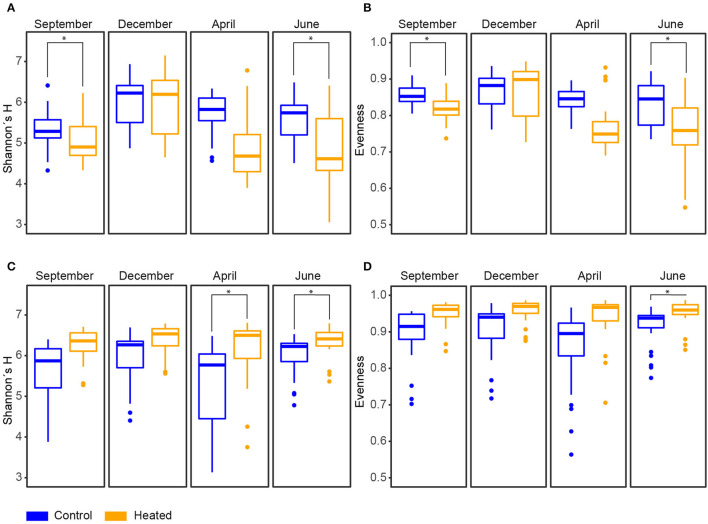
Diversity of microbial communities in bottom water and sediment over different seasons in the heated and control bays. Heated (orange) and control (blue) bay diversity indices for the four sampling occasions between September 2017–June 2018 (all *n* = 191), showing **(A)** Shannon's H diversity and **(B)** evenness based on Shannon's H diversity index for bottom water samples (sample “X2061” was excluded due to the small amount of reads; Supplementary Table 1). **(C)** Shannon's H diversity and **(D)** evenness based on Shannon's H diversity index for sediment samples (samples “X51” and “X11” were excluded; Supplementary Table 1); Significant differences between bays are marked with an asterisk.

The Alpha diversity and evenness indices in the BW communities showed a significantly higher Shannon's H diversity in the control bay compared to the heated bay [mean ± s.d., a 5.2 ± 0.9 heated bay vs. a 5.7 ± 0.6 control bay, ANOVA, *F*_(1, 14)_ = 7.3, *p* = 0.0173] and evenness [0.8 ± 0.1 vs.0.9 ± 0.1, *F*_(1, 14)_ = 16.7, *p* = 0.001; Figure 3, Supplementary Figure 1, Supplementary Table 3]. Overall, more ASVs were found within the control bay (Chao1, mean, 720 ± 402.7 vs. 861.5 ± 321.2; Supplementary Table 1). The opposite pattern was observed in the 0–1-cm SED with an insignificant increase in the heated bay Shannon's H diversity (6.3 ± 0.5 vs. 5.7 ± 0.8) compared to the control bay, significantly higher number of ASVs [761.1 ± 162.8 vs. 630.8 ± 193.9, ANOVA, *F*_(1, 14)_ = 4.5, *p* = 0.05], and more equal distribution (0.9 ± 0.04 vs. 0.8 ± 0.1; Figure 3, Supplementary Figure 1, Supplementary Tables 1, 3).

### Microbial Communities in Sediment and Bottom Waters

16S rRNA gene amplicon sequencing of the BW microbial communities showed that the most abundant phyla in both bays were *Actinobacteria* [mean of relative abundance of total reads (%) of all sampling occasions: 29.9 ± 7.9 heated bay vs. 28.4 ± 6.3% control bay], *Proteobacteria* (21.8 ± 4. vs. 23.2 ± 3.2%), *Bacteriodota* (19.8 ± 6.3 vs. 13.2 ± 3.4%), and *Cyanobacteria* (11.3 ± 4.9 vs. 13. ± 5.2%; Supplementary Figure 6). The most abundant SED phyla in both bays were similar as found in the BW with *Proteobacteria* (27.7 ± 4.1 vs. 25.9 ± 3.1%), *Cyanobacteria* (5.2 ± 3.8 vs. 11.1 ± 8.2%), *Bacteriodota* (13.5 ± 3.5% vs. 16.5 ± 4.4%), and *Desulfobacteriodota* (9.8 ± 3.7 vs. 11.2 ± 2.1%; Supplementary Figure 6). Similarity of percentages (SIMPER) analysis showed 56.1% (BW) and 53.3% (SED) of overall dissimilarity between communities of both bays on the order level. The most likely taxa responsible for the differences between the BW communities were, e.g., *Flavobacteriales* (8.1% average contribution of dissimilarity), *Synechococcales A*. (6.1%), *Microtrichales* (3.9%), *Betaproteobacteriales* (3.6%), and *Nanopelagicales* (3.4%) (Table 3, Supplementary Table 4). Those taxa make up 39.2 ± 11.4% within the heated (37.7 ± 12.1% of the community significantly differentially abundant ASVs) and 36.2 ± 7.8% (33.7 ± 8.1% significant ASVs) within the control bay of the relative abundance of the whole bacterial community dataset. SIMPER analysis of the SED communities on the order level showed that *Cyanobacteriales* were most likely one of the main contributors to differences with 3.85%, followed by *Betaproteobacteriales* (3.5%), *Desulfobacteriales* (3.3%), *Anaerolineales* (2.8%), and *Flavobacteriales* (2.7%; Table 3, Supplementary Table 4). These top five orders make up 16.9 ± 7.8% (9.2 ± 3.9% significant ASVs) and 21.9 ± 5.8% (13.8 ± 5.3% significant ASVs) of the relative abundance in the heated and control bays, respectively.

**Figure 4:**
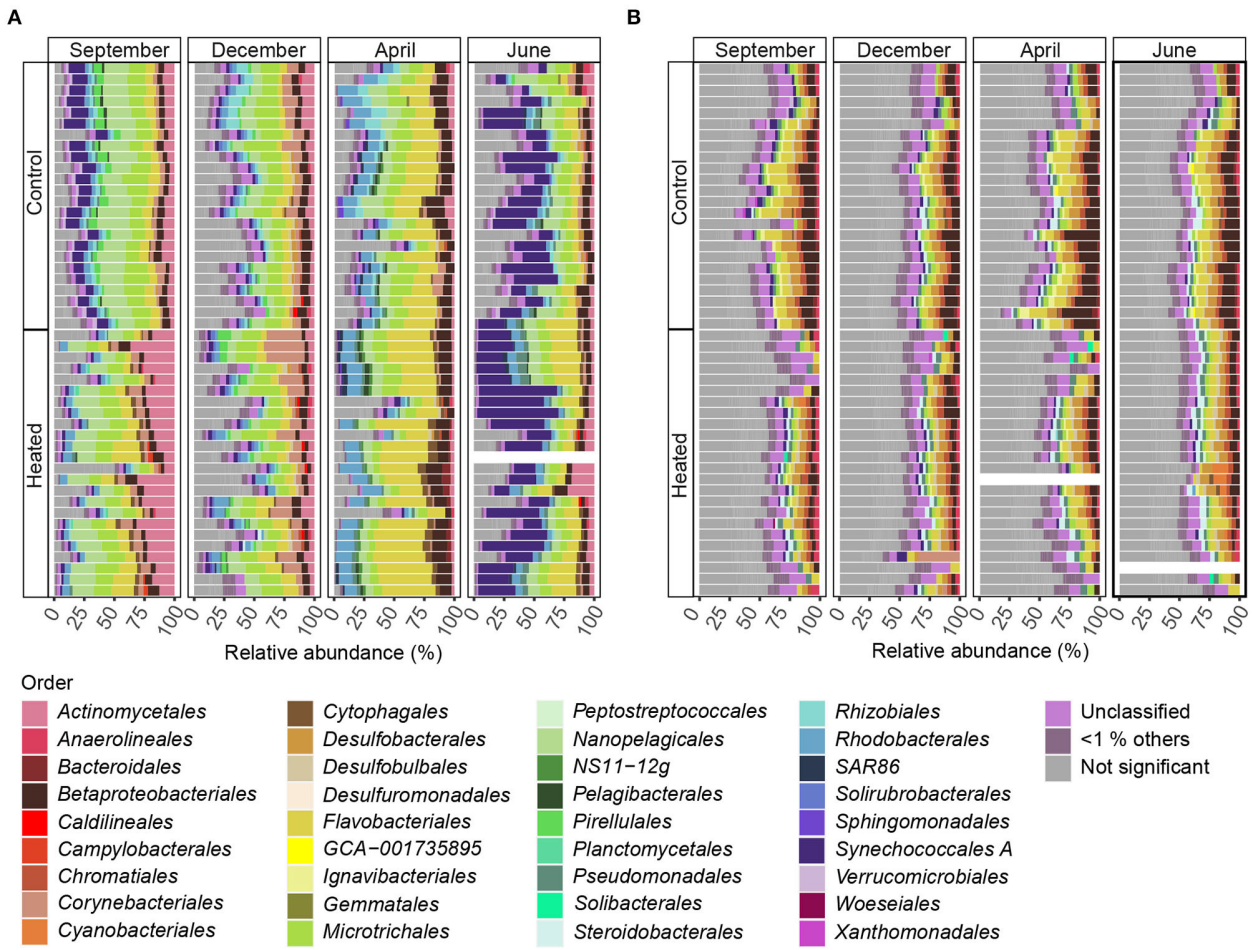
Differentially abundant ASVs summarized means on the order level for bottom water and sediment. Stacked bars of significant differentially abundant ASVs between the heated and control bays (both *n* = 191) for the different sampling months for **(A)** bottom water and **(B)** sediment. The x-axes show the relative abundance summed to 100%, while the y-axis shows the bays with rows representing sampling sites (*n* = 8) and replicates (*n* = 3). Shown are significant differentially abundant ASVs with summarized mean on the order level above 1% relative abundance (significant ASVs below 1% relative abundance are grouped as “ <1% others” and insignificant differentially abundant ASVs between bays as “not significant.” Samples with low read numbers have been excluded (see Materials and Methods). The stacked bars are read from right to left.

Significantly differentially abundant ASVs summarized and annotated on the order level (with >1% relative abundance per sample) within BW samples of the heated and control bays showed a strong seasonal influence on bacterial community composition (Figure 4, Supplementary Table 5). Between both analyzed bays, BW samples comprised a slightly higher proportion of ASVs that differed significantly in abundance compared to SED communities. On average, 80.8% of the ASVs were significantly and differently abundant in the heated bay, while 79.4% were significant in the control bay (Figure 4). Within the high variation of bacterial community composition, the order *Nanopelagicales* (mean, a 6.6 ± 2.4% heated bay vs. a 7.8 ± 2.6% control bay) were more differentially abundant in the control bay compared to *Flavobacteriales* (a 12.1 ± 5.8% heated bay vs. a 6.4 ± 2.9% control bay) that were higher in the heated bay (Figure 4, Supplementary Table 6).

**Table 3:** SIMPER analysis.

	**Order**	**Average**	**sd**	**Ratio**	**Average (heated)**	**Average (control)**	**Cumsum**
Bottom water	Flavobacteriales	0.081	0.075	1.075	14,761.83	7,837.11	0.144
	SynechococcalesA	0.060	0.084	0.723	7,245.02	6,174.05	0.253
	Microtrichales	0.039	0.031	1.243	8,144.6	6,993.55	0.323
	Betaproteobacteriales	0.036	0.031	1.139	5,854.63	6,054.09	0.388
	Nanopelagicales	0.033	0.029	1.157	6,279.87	5,478.70	0.448
	Actinomycetales	0.030	0.038	0.795	5,617.13	2,441.04	0.503
	Rhodobacterales	0.029	0.024	1.232	5,799.23	3,204.66	0.556
	Corynebacteriales	0.022	0.033	0.653	3,905	2,171.70	0.595
	Pirellulales	0.015	0.017	0.887	2,454.71	1,999.07	0.624
	Anaerolineales	0.014	0.015	0.914	1,526.43	1,870.20	0.649
Sediment	Cyanobacteriales	0.039	0.044	0.880	1,656.31	3,899.96	0.072
	Betaproteobacteriales	0.035	0.025	1.422	2,971.64	4,359.64	0.138
	Desulfobacterales	0.033	0.025	1.334	2,519.34	3,924.72	0.200
	Anaerolineales	0.028	0.021	1.327	2,546.54	2,989.63	0.254
	Flavobacteriales	0.027	0.023	1.209	2,147.79	3,261.61	0.305
	Chromatiales	0.023	0.018	1.247	1,892.33	2,444.72	0.348
	Bacteroidales	0.021	0.016	1.330	1,675.43	2,338.38	0.387
	Pirellulales	0.019	0.018	1.096	1,900.12	1,405.74	0.424
	Pseudomonadales	0.019	0.016	1.200	1,864.35	1,856.36	0.459
	Desulfobulbales	0.016	0.014	1.127	1,273.75	1,658.30	0.489

*An overview of the top 10 taxa responsible for changes between the bays. Shown are the community on the order level; average contribution to overall Bray-Curtis dissimilarity; standard deviation; the ratio of average-to-standard contribution; average of abundance of each group (a and b); and the cumulative contributions. SIMPER was calculated for comparing the dissimilarities between the heated and control bays of bottom water and sediment samples separately*.

In contrast, surface SED communities did not show strong seasonal influence on significant differentially abundant ASVs (mean summarized on the order level >1% relative abundance; Figure 4), and the number of non-significant differentially abundant ASVs was, on average, 23.3% higher within sediment communities (19.9% BW vs. 43.3% SED). The main abundant taxa were *Desulfobacteriales* (1.82 ± 1.00% and 3.10 ± 1.07%), *Flavobacteriales* (2.3 ±0.9 vs. 3.7 ± 2.1%), and *Betaproteobacteriales* (2.4 ± 1.5 vs. 4.7 ± 2.6%) that had higher relative abundances in the control vs. the heated bay (Figure 4). *Betaproteobacteriales* were significantly positively correlated with phosphate (Pearson correlation, *p* = 0.02, *r* = 0.26), sulfate (*p* = 0.002, *r* = 0.29), and organic matter (*p* = 0.0002, *r* = 0.35) while decreasing with increasing temperature (*p* = 0.04, *r* = 0.22; Figure 4, Supplementary Figure 7, Supplementary Table 6). Furthermore, *Flavobacteriales* were positively correlated with sulfate (*p* = 0.03, *r* = 0.21), while *Desulfobacteriales* positively correlated to phosphate (*p* < 0.001, *r* = 0.32), organic matter (*p* = 0.02, *r* = 0.23), and temperature (*p* = 0.003, *r* = 0.28; Figure 4, Supplementary Figure 7, Supplementary Table 6).

### Linking Microbial Communities to Geochemical Fluctuations

**Figure 5:**
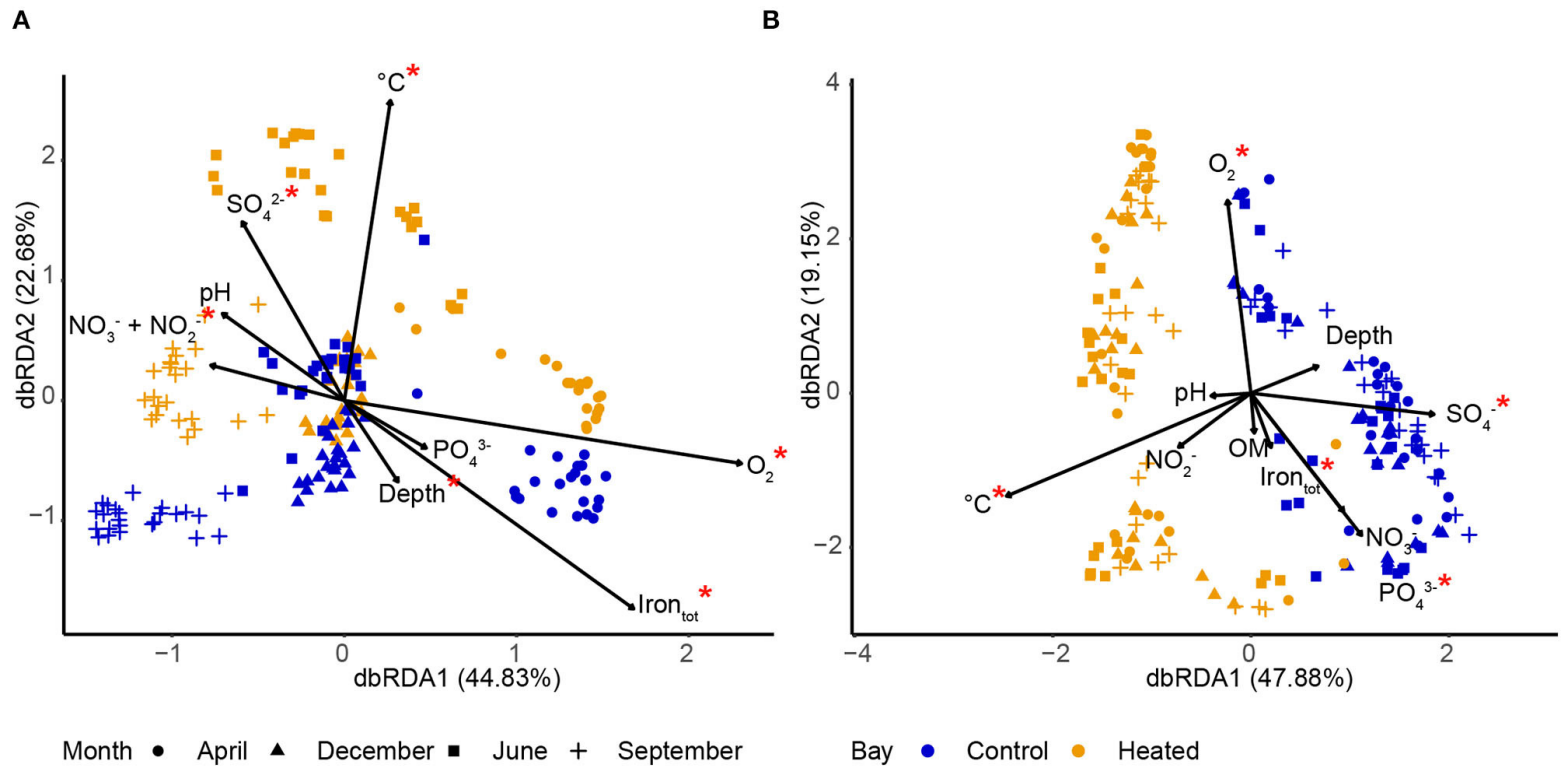
Distance-based redundancy analysis (db-RDA) of communities and environmental variables in bottom water **(A)** and sediment **(B)** in the heated (orange) and control (blue) bays. Constrained ordination using samples from 0 to 1 cm sediment of three replicates for each site (*n* = 8) on each sampling month (*n* = 4) in each bay (*n* = 2), giving a total of 192 samples. The input ASV data were based on relative abundances (%). Significantly different (*p* < 0.05) bottom water **(A)** and sediment pore water **(B)** environmental variables are marked with a red asterisk (statistics detailed in Supplementary Table 3), and the degree of variation explained by the first and second axes is indicated.

Bacterial communities in BW were clustered by the different sampling months. The fluctuations in geochemical parameters included temperature (permutation test, ANOVA, *F* = 14.12, *p* = 0.001) and temperature-related parameters, including O_2_ (*F* = 17.75, *p* = 0.001), ${\text{NO}}_{3}^{-}$/${\text{NO}}_{2}^{-}$ (*F* = 5.06, *p* = 0.001), total Fe (*F* = 6.88, *p* = 0.001), and ${\text{SO}}_{4}^{2-}$ concentrations (3.39, *p* = 0.003; Supplementary Table 3), associated with the contrasting communities (Figure 5). Microorganisms in BW in both bays clustered together significantly on the first axis (permutation test, ANOVA, *F* = 27.09, *p* = 0.001) according to the sampling time (season) and on the second axis according to the different bays (*F* = 13.62, *p* = 0.001; Figure 5, Supplementary Table 3). This indicated that short-term changes, such as seasonal variation, were the dominant drivers for community composition compared to long-term changes of increased temperature and prolonged warming (Figure 5). The communities of both bays were positively related with high O_2_ concentrations in April and showed lower O_2_ during the rest of the year, indicating high-water circulation-replenished O_2_ during spring (Figure 5). Potential influences on microbial community composition in the heated bay could also be the higher ${\text{SO}}_{4}^{2-}$concentrations in June in the heated bay and lower ${\text{NO}}_{3}^{-}$/${\text{NO}}_{2}^{-}$ (in combination) in April plus high concentrations in September. Finally, temperature was positively related with the heated bay communities (Figure 5).

Seasonal fluctuations likely did not substantially affect SED communities as there was a strong clustering on the first axis due to the different bays (permutation test, ANOVA, *F* = 9.51, *p* = 0.001; Figure 5, Supplementary Table 3), with no strong seasonal distribution between the bays, indicating that long-term changes, such as prolonged increased temperature, were the dominant drivers. The main influencing factors shaping the microbial communities within the different sampling months were temperature (permutation test, ANOVA, *F* = 4.59, *p* = 0.001), O_2_ (*F* = 3.43, *p* = 0.002), ${\text{SO}}_{4}^{2-}$ (*F* = 3.24, *p* = 0.004), ${\text{PO}}_{4}^{3-}$ (*F* = 1.83, *p* = 0.043), and total Fe (*F* = 1.91, *p* = 0.052: Figure 5, Supplementary Table 3). The data indicated that long-term increased temperatures of spatial factors played an important role in shaping the microbial communities in the SED surface (Figure 5).

### Seasonal Response of Microbial Communities in Sediment and Bottom Waters

**Figure 6:**
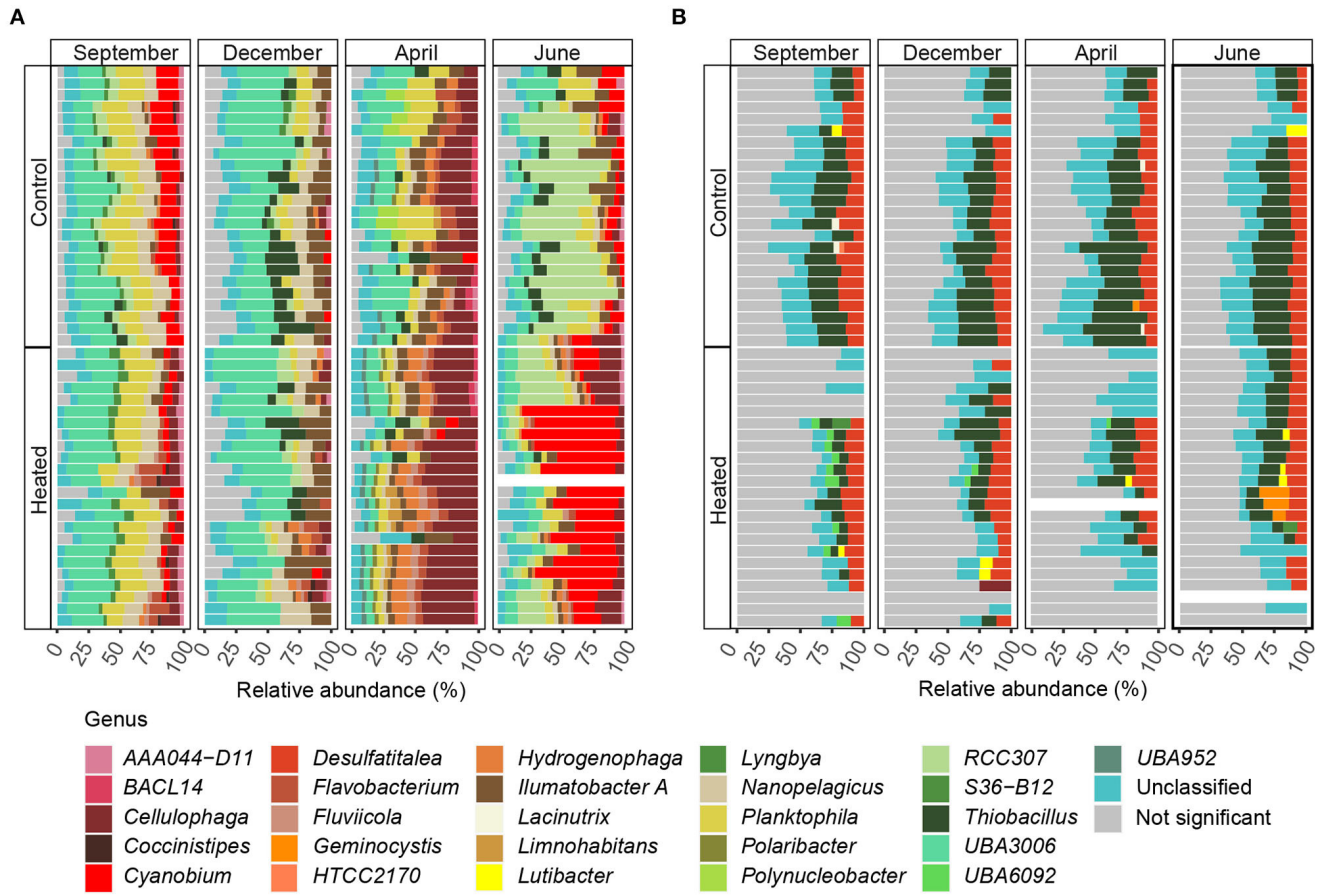
Differentially abundant ASVs summarized mean on the genus level within the top five orders responsible for shaping BW **(A)** and SED **(B)** bacterial communities according to SIMPER analysis. Stacked bars of significantly differentially abundant ASVs responsible for differences between the heated and control bays at the different sampling months (*n* = 4). The mean relative abundance was calculated on the genus level, and taxa below 1% relative abundance were excluded. Shown are the different sampling sites plus replicates (*n* = 24) per bay and month. Nonsignificant differentially abundant ASVs were grouped together. Samples with low read size were excluded (see Materials and Methods). The stacked bars are read from right to left.

The BW community Shannon's H Alpha diversities between bays were significantly different in June (ANOVA, pairwise comparison, *p* < 0.01) and September (*p* < 0.01; Figure 3, Supplementary Table 3). Within the BW, the order *Actinomycetales* peaked in September (mean relative abundance, a 16.2 ± 5.4% heated bay vs. a 5.5 ± 1.9% control bay) with the highest abundance within the heated bay positively correlated with temperature (Pearson correlation, *p* = 0.0001, *r* = 0.36) and negatively with oxygen (*p* = 0.001, *r* = −0.26). In addition, *Nanopelagicales*-relative abundance increased in both bays in September (a 12.4 ± 3.4 heated bay vs. a 15.4 ± 3.5% control bay) and was positively correlated with higher temperature (*p* < 0.0001; Supplementary Table 6). The main contributor to this increased relative abundance in September was the genus *Planktophila* (mean ± s.d. of significant ASVs summarized on the genus level, 5.9 ± 1.5 vs. 7.6 ± 2.6%) that had higher abundances in the control bay in December (1.8 ± 0.9%) compared to the heated bay (0.9 ± 0.5%; Figure 6). Heated-bay BW showed a rapid change in *Cyanobacteria* compared to the control bay, while, in June, the cyanobacterial abundances were higher in the heated bay (23 ± 12.6%); they dropped rapidly in September (1.8 ± 0.5%), and stayed low in December (1.9 ± 0.8%). The *Cyanobacteria* order *Synechococcales A* peaked in June with positive correlation with temperature (*p* < 0.0001, *r* = 0.36) plus sulfate (*p* < 0.0001, *r* = 0.39) and higher relative abundance in the heated compared to control bay with mean relative abundances of 23 ± 12.6 vs. 15.7 ± 9% (Figure 4, Supplementary Figure 7). These high abundances were mainly due to the genus *Cyanobium* (16.6 ± 13.5%). Notably, the dominant bloom in June in the control bay was *RCC307* (13.1 ± 9.3%) within the *Synechococcales A* order (Figure 6). The second bloom in September was dominated by *Cyanobium* in both bays but already decreased in the heated bay (1.3 ± 0.4%) compared to the control bay (5.9 ± 1.9%), indicating that peak blooms may have occurred earlier within the heated bay. With oxygen-rich spring BW, the peak in abundance of, e.g., *Rhodobacteriales* (positively correlated with oxygen, *p* < 0.0001, *r* = 0.41) and *Flavobacteriales* (*p* < 0.0001, *r* = 0.42; Figure 4, Supplementary Figure 7), was observed in both bays in April. The main contributors to the increased abundance were the genus *Cellulophaga* within the order *Flavobacteriales*, with higher abundances within the heated bay (14.4 ± 6.3%; Figure 6). On the order level, *Betaproteobacteriales* showed similar peak abundances in both bays within April (7.6 ± 2.4 vs. 5.2 ± 1%), while the genus *Thiobacillus* showed high abundance within the control bay in December (2. ± 1.3%) compared to the heated bay (0.8 ± 0.7%; Figure 6).

For the sediment, significantly higher Shannon's H Alpha diversities could be observed in April (ANOVA, pairwise comparison, *p* < 0.05) and June (*p* < 0.05; Figure 3, Supplementary Table 3) in the heated bay. Overall, the proportion of abundant microorganisms was more stable with the changing season but showed differences between the bays. For example, the order *Anaerolineales* showed overall higher concentrations in the heated (mean ± s.d. 1.2 ± 0.4%) compared to the control bay (0.9 ± 0.5%), while their abundance peaks in September (1.7 ± 0.8%) and June (1 ± 0.5%). The main contributor of the peak in September was the genus *UBA6092* (0.81 ± 0.42%), which did not occur in higher abundance in the control bay (0.4 ± 0.3%). A similar pattern was observed within *Rhodobacterales* that had an overall low abundance in both bays but was, on average, 37.5% higher in the heated bay (Figure 4). On the other hand, the control bay showed a higher significant differential abundance of *Betaproteobacteriales* over all seasons compared to the heated bay. The main contributor was the genus *Thiobacillus*, which was the dominant taxon in all sampling sites and seasons within the control bay (4.8 ± 2.6%). Within the control bay, the order *Desulfobacteriales*, which was, on average, 28.6% higher abundant compared to the heated bay with peaks in September (3.5 ± 1.3%) and June (3.2 ± 1.1%), as well as *Flavobacteriales*, with an average of 32.3% higher abundance, was found.

In summary, cyanobacteria RCC307 and *Cyanobium* increased in relative abundance within the control bay bottom waters in the June and September samples, respectively. Within the heated bay, there was an increase of bottom water cyanobacteria-relative abundance in June due to the genus *Cyanobium* that decreased to a low-relative abundance in September. Furthermore, surface sediment microbial communities were more stable in composition over the year, showing overall higher relative abundances of *Anaerolineales* and *Rhodobacteriales* in the heated bay compared to higher relative abundances of *Thiobacillus* in the control bay.

### Metabolic Responses in Sediment and Bottom Waters Communities

**Figure 7:**
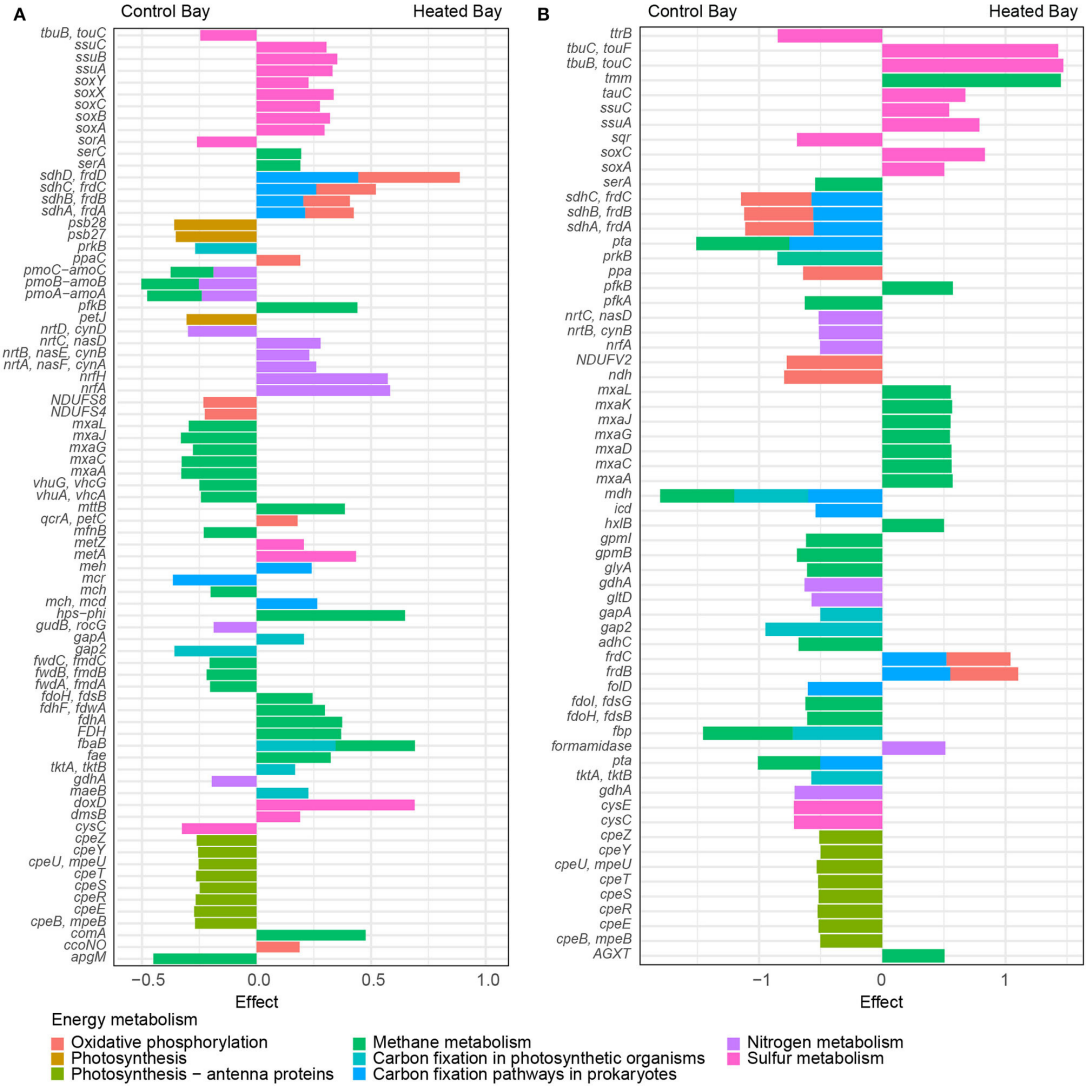
Differential abundance of predicted metabolic response of highly abundant ASVs in bottom water and sediment over all sampling times. **(A)** Bottom water and sediment **(B)** of significant (*p* < 0.05) differentially abundant genes between the heated and control bays predicted using the tool PICRUSt2.0. The colors show the different energy metabolism the genes are involved in based on KO identifiers. The axis shows the effect size of the significant differential abundances in the heated bay (positive values) and the control bay (negative values). The effect size shows the ratio between the abundance of the two conditions, similar to a Fold Change.

PICRUSt2.0 predictions of BW metabolic responses (based on KO-identifiers) of the differentially abundant ASVs between the bays showed a significant increase in sulfur and nitrogen metabolism-associated genes within the heated bay, while a higher number of significantly different genes associated within photosynthesis pathways were found in the control bay (Figure 7, Supplementary Table 7). In addition, a significant increase of predicted genes associated with sulfur oxidation (SOX-system) and nitrogen metabolism-associated genes were found in the heated bay (Figure 7). Furthermore, nitrate and potentially nitrite uptake genes were significantly higher abundant in the heated bay and likely associated with *Cyanobacteria* (Flores et al., 2005). Genes associated with the dissimilatory nitrate reduction to ammonium (DNRA, genes *nrfA* and *nrfH*) were significantly more abundant in the heated bay (Figure 7, Supplementary Table 7). The control bay BW showed a different picture with the photosynthesis associated genes *psb28* and *psb27* significantly more abundant than the heated bay, which were likely associated with *Cyanobacteria* blooms (Figure 7). Further oxygenic photosynthetic light harvesting system-associated genes, including *cpeZ, cpeY*, and *cpeU*, had significantly higher relative abundance in the control bay (Figure 7, Supplementary Table 7).

Prediction of genes involved in SED community metabolism (Supplementary Table 7) showed a slightly shifted pattern within energy metabolism. While nitrogen metabolism genes were significantly differently abundant in the heated bay BW, there was no significant difference found within the heated bay sediment (Figure 7). Instead, genes related to the sulfur and methane metabolism were the main abundant predicted genes in the heated bay. In contrast, in total, more genes were significantly differently abundant in the control bay compared to the heated bay, including those coding for oxidative phosphorylation, photosynthesis, and nitrogen metabolism (Figure 7, Supplementary Table 7). Within the heated bay, sulfur oxidation *soxAC* plus conversion of methanol to formaldehyde within methane oxidation (*mxaLKJGDCA*) genes were significantly more abundant (Figure 7). In comparison, photosynthesis genes (*cpeZYUTSREB*) plus *gapA, gap2*, and *tktB* carbon fixation genes from photosynthetic organisms were significantly higher in the control bay (Figure 7). The higher abundance of *Cyanobacteria* within the control bay SED surface (Supplementary Figure 6) was further strengthened by the predicted significantly differentially abundant *nrtC* and *nrtB* genes for nitrate/nitrite uptake (Figure 7). Finally, sulfur metabolism played a minor role in the control bay differentially abundant genes, while methane oxidation *gpmLB* was relatively more abundant (Figure 7).

## Discussion

While climate change effects strongly depend on the location (Gruner et al., 2017), some of the most vulnerable regions worldwide will be coastal areas (Bini and Rossi, 2021). In addition, coastal marine ecosystems are especially stressed due to dense human populations living near the coasts, resulting in increased inputs of waste and nutrients into the waters that will exacerbate climate change-related effects, such as increasing temperature, decreased oxygen concentrations, and acidification (Reusch et al., 2018). The interest in climate change and global warming has been huge to this day, but most studies are short-term laboratory-based simplifications of the real world to control for various parameters, which would make the interpretation of experiments otherwise more complicated (Forsman et al., 2016). This study used a natural fluctuating system, which has been warmed for more than 50 years, and gave the opportunity to investigate on large-scale, long-term, and complex multi-species interactions in the closest-to-nature-possible conditions.

The bacterial composition found in the control and heated bay surface sediment and bottom waters showed similarity to community compositions found in other coastal Baltic Sea bays (Broman et al., 2015), while the relative abundance varied. For example, *Actinobacteria* have a preference for higher temperatures (Berner et al., 2018), and a small but significant increase in relative abundance of small-sized bacteria, such as *Rhodoluna* (Hahn, 2016), was observed within the heated-bay bottom waters. Small bacteria can have a metabolic advantage at higher temperatures compared to larger microorganisms as the increased surface to the volume ratio promotes, e.g., gas exchange and nutrient uptake (Hessen et al., 2013). The prolonged warmer waters favored the growth of small-sized autotrophic picophytoplankton cells (Rasconi et al., 2015), which are further discussed below. These observations could give either a first hint that warmer water temperatures can increase the abundance of smaller-sized microorganisms in the future or, alternatively, natural environmental fluctuations occurred.

Bacterial communities in coastal bottom waters are continuously exposed and shaped by seasonal fluctuations, while climate change is expected to suspend these well-defined seasonal patterns (Visser and Both, 2005). A further effect of rising temperatures on coastal ecosystems will be increased phytoplankton blooms (Andersson et al., 2015). The elevated Cyanobacteria summer (June) bloom has competitive advantages at higher temperatures (Lurling et al., 2018) in the heated bay water and was dominated by the common brackish water genus *Cyanobium*. In contrast, another picocyanbacteria, *RCC307* (Scanlan et al., 2009), were dominant in June within the control bay, potentially due to the lower salinity (Kim et al., 2018). While picocyanobacteria (i.e., <2 μm in size belonging to the *Synechococcus/Cyanobium* group) are typically non-bloomers, they can still form dense populations (Steffen et al., 2012) during the summer/autumn (Kuosa, 1991) and are one of the most abundant Cyanobacteria found in Baltic Sea dead zone sediments (Broman et al., 2021). Picocyanobacteria dynamics are dependent on nutrient concentrations, temperature, and light conditions (Otero-Ferrer et al., 2018), with ${\text{NH}}_{4}^{-}$as the preferred nitrogen resource for *Synechococcus/Cyanobium* (Moore et al., 2002), especially at higher temperatures when assimilation of ${\text{NH}}_{4}^{+}$ is greater than ${\text{NO}}_{3}^{-}$ (Glibert et al., 2016) that could potentially explain the relative dominance of *Cyanobium* in the two bays. The observation of higher abundance of Cyanobacteria in June but lower abundances in September compared to the control bay might suggest an earlier summer bloom within the heated bay Cyanobacteria, which would be consistent with a 32-year time series, showing lengthened Baltic Sea-warmth-shifted Cyanobacteria blooms from August to July (Kahru et al., 2016). The potential earlier blooming in the heated bay Cyanobacteria and, therefore, low-relative abundance in late summer (September) likely also led to the observed lower organic-matter concentration and probably resulting in decreased *Desulfobacteriales* (Lefèvre et al., 2011) and *Flavobacteriales* that play a vital role within initial organic matter degradation (Bissett et al., 2008). Finally, the increased relative abundance of algae-associated genera like *Cellulophaga* (Johansen et al., 1999) within the heated bay may suggest an increased algal bloom during April and June. Elevated temperatures will likely lead to a shift and prolonged blooming of especially smaller-sized picocyanobacteria in the future, with higher aggregation of biomass sinking to the sediment surface, resulting in accelerated bacterial respiration rates and exacerbated dead zones.

Classical niche theory states that temporal environmental heterogeneity harbors more species (Tamme et al., 2010) with large temperature fluctuations, resulting in coexistence of multiple species becoming dominant at various times of the year (Gruner et al., 2017). Therefore, decreased Alpha diversity within the heated bay bottom waters was likely due to suspension of seasonal patterns, with prolonged warming into colder months, having a particularly strong effect (Gutiérrez et al., 2018). An opposite trend was observed within surface-sediment communities, where a more homogenous distribution and higher number of species were found within the heated bay compared to the control bay that supported previous findings (Seidel et al., 2022). No significant differences were observed in oxygen concentrations and sediment OM (%) between the bays, indicating that the changes in microbial communities likely reflected a temperature effect. Nevertheless, the resulting effect was similar to selection for sulfate-reducing bacteria in, e.g., the Caspian Sea where high nutrient input led to oxygen-depleted areas, raising the redox layer toward the sediment surface in a similar manner to that with increasing temperature (Hicks et al., 2018) and leading to greater species richness. Therefore, future climate change-related warming is likely to diminish bacterial diversity in benthic waters on one hand, while, on the other hand, the effect of higher temperatures in sediments will result in higher diversities of microorganisms in surface sediments due to the thinning of geochemical zones.

The higher phytoplankton growth discussed above could potentially have resulted in increased biomass, sinking down to the seafloor (at sampling sites further from the water outlet) in, e.g., June, leading to enhanced microbial activity and respiration rates in coastal surface sediments (Nydahl et al., 2013). Higher organic matter content has been previously observed in the heated bay (Seidel et al., 2022). Potentially elevated sedimentation rates leading to decreasing O_2_ concentrations and, therefore, lower binding of ${\text{PO}}_{4}^{3-}$ to Fe oxides were likely coupled to the generally lower phosphate concentrations in the heated bay (Carstensen et al., 2020). The observed metabolic response prediction of the heated-bay 16S rRNA gene amplicons within sediments confirmed previous findings of a potential increase in microbial activity and, therefore, reduced levels of anaerobic electron acceptors (Seidel et al., 2022). The predicted metabolic response data also supported the previous observation of increased microbial activity, potentially enhancing oxygen consumption closer to the sediment-water interface (Seidel et al., 2022), preventing oxygen penetration into the sediment, and the use of alternative anaerobic electron acceptors (de Klein et al., 2017; Jørgensen et al., 2019). The PICRUSt2 analysis predicted increased gene copy numbers coding for anaerobic processes, such as nitrogen and sulfur cycling taxa, and transcripts in the heated-bay surface sediment, which was in accordance with previous RNA transcript data from the two bays (Seidel et al., 2022). For example, the predicted *nif* D (nitrogen fixation) gene copy number was higher within the taxa *Sedimenticolaceae* and *Desulfocapsaceae* that both had higher relative abundances in the heated bay compared to the control bay. Furthermore, within the sulfur cycle prediction for the *fcc*B (sulfur oxidation) gene, increased relative abundances of *Sedimenticolacea*e and *Woseiaceae* were detected in the heated bay compared to the control bay. These results further strengthened the PICRUSt2 outcome. In addition, decreased sulfate concentrations in April and September and the overall increase in predicted gene copies related to thiosulfate (*sox*A/C) and methanol (*mxa*ACDGJKL) oxidation suggested higher sulfide and methane metabolism closer to the sediment surface. Elevated sulfur cycling supported the previously reported higher heated-bay sediment sulfate reduction rates and increased transcripts related to sulfate reduction, including *dsvB* and *aprAB* (Seidel et al., 2022). Sulfate reduction accounts for ~50% of the organic carbon mineralization in coastal sediments (Jørgensen, 1982) and is accompanied by sulfide oxidation within the cryptic sulfur cycle. However, sulfate is not often depleted close to the sediment surface, as it penetrates from the seawater or the resulting sulfide is directly re-oxidized (Canfield et al., 1992; Luther et al., 2011). The prolonged reduction of sulfate into the autumn (September) within the heated bay may have led to the lower concentration of sulfate. Sediments harbor large diversities of sulfide-oxidizing microorganisms, such as *Thiobacillus, Beggiatoa, Thermothrix, Thiothrix, Sulfurimonas, and Sulfurovum* (Behera et al., 2014; Broman et al., 2017a,b), and in the previous study (Seidel et al., 2022) the observed increase in RNA transcripts was ascribed to the *Thiobacillus* in heated-bay sediments (that did not show significant differential higher relative abundances within this study). The findings of the present study were in accordance with previously observed results of the shallowing of geochemical layers due to long-term increased temperatures and could potentially suggest compressing of redox layers closer to the sediment surface.

## Conclusion

Coastal ecosystems subjected to ~50 years of temperature increase showed potential disturbance of classical seasonal fluctuations, resulting in likely enhanced growth and earlier appearance of picocyanobacteria during the summer. This influenced and decreased Shannon's H diversity of microbial communities in bottom waters and changed accompanying environmental factors, such as nutrient concentrations. The cascade of fluctuations within bottom waters likely led to higher oxygen consumption in the sediment surface, resulting in an increase of anaerobic processes closer to the sediment surface and higher Shannon's H diversities. This study used a long-term model system with natural fluctuations to provide support for climate change-related shifts toward smaller-sized microorganisms, altered seasonality in cyanobacterial blooms, reduced microbial diversities, and, potentially, higher respiration rates within future climate change coastal waters.

## Data Availability Statement

The 16S rRNA gene sequencing data are available on the NCBI database under BioProject PRJNA783808. The code to generate the figures and statistical testing can be found on https://github.com/laseab/CC_cBS_pico.

## Author Contributions

MD, SH, AF, LS, and EB designed the study. LS, EB, and MS collected the samples. LS, MS, WH, EN, and VS analyzed the samples. LS, MD, AF, SH, and ST evaluated the data. LS and MD drafted the manuscript. All authors read and approved the final manuscript version.

## Funding

MD thanks the Swedish Research Council for Sustainable Development, Formas (contract FR-2020/0008), AF thanks the Swedish Research Council, Vetenskaprådet (contract 2020-03519), SH acknowledges The Crafoord Foundation (Grant No. 20170539), and AF acknowledges the Magnus Bergvalls Stiftelse (Grant No. 2019-03116) for financial support. The computations were enabled by resources (SNIC 2021/22-628 and SNIC 2021/6-256) provided by the Swedish National Infrastructure for Computing (SNIC) at UPPMAX at Uppsala University, partially funded by the Swedish Research Council through grant agreement No. 2018-05973.

## Conflict of Interest

The authors declare that the research was conducted in the absence of any commercial or financial relationships that could be construed as a potential conflict of interest.

## Publisher's Note

All claims expressed in this article are solely those of the author and do not necessarily represent those of their affiliated organizations, or those of the publisher, the editors and the reviewers. Any product that may be evaluated in this article, or claim that may be made by its manufacturer, is not guaranteed or endorsed by the publisher.
