# Supplementary material for: Long-Term Warming of Baltic Sea Coastal Waters Affects Bacterial Communities in Bottom Water and Sediments Differently
Source: Front Microbiol. 2022 Jun 10;13:873281. doi: 10.3389/fmicb.2022.873281 (PMC9226639; doi:10.3389/fmicb.2022.873281)
Supplement: Supplementary file 8 [file Data_Sheet_1.docx]

Supplementary Material

**Long-term Warming of Baltic Sea Coastal Waters Affects Bacterial Communities in Bottom Water and Sediments Differently**

Laura Seidel, Elias Broman, Magnus Ståhle, Emelie Nilsson, Stephanie Turner, Wouter Hendrycks, Varvara Sachpazidou, Anders Forsman, Samuel Hylander, and Mark Dopson

**Supplemental Table 1.** Sample information and community composition. Meta table for each sample including sampling times, locations, and environmental variables. Additionally, overview about the sequenced community composition on order level (count data) for the bottom water and sediment samples.

**A separate Excel file has been uploaded.**

**Supplemental Table 2.** Sequencing information. Information of the samples sequenced including DNA concentration, sequencing platform, read length, and million reads sequenced, as well as the amount of filtered reads left after each step of the DADA2 pipeline.

**A separate Excel file has been uploaded.**

**Supplemental Table 3.** Statistical tests. Comparison of bays community composition of the bottom water (BW) and sediment (SED) samples, respectively. Included are also linear mixed model statistical testing to compare environmental variables and diversity indices of the bays and sampling times, as well as anova like permutation tests to test which environmental variables were significant explaining differences between bacterial communities. The various statistical analysis can be found within the different sheets of the excel table.

**A separate Excel file has been uploaded.**

**Supplemental Table 4.** SIMPER analysis on order level of bottom water and sediment samples. Overview about the contribution of dissimilarity between the bacterial communities of the heated and control bay on order level using SIMPER. Shown are the community on order level; average contribution to overall Bray-Curtis dissimilarity; standard deviation; ratio of average to standard contribution; average of abundance of each group (a & b) and the cumulative contributions. SIMPER was calculated for comparing the dissimilarities between the heated and control bay of bottom water and sediment samples separately.

**A separate Excel file has been uploaded.**

**Supplemental Table 5.** Differential abundance analysis of the 16S rRNA gene amplicon ASVs of the tested bottom water (BW) and sediment samples (SED). Analysis was done on ASVs for each month comparing bays using the contrast function within the DESeq2 package; ASV= amplicon sequence variants, baseMean, mean Control bay, mean Heated bay, log2FoldChange, lfcSE= Standard Error of LogFoldChange measured, stats, *p*-value; padj= adjusted p-value with Benjamin Hochberg correction; kingdom, phylum, class, order, family, genus, and species.

**A separate Excel file has been uploaded.**

**Supplemental Table 6.** Pearson Correlation. Correlation analysis of significant differential abundant ASVs > 1 % relative abundance within bottom water (BW) and sediment samples (SED). The counts of the ASVs were summarized on order level and *clr* transformed before the analysis was taken place. Order= Parameter 1, Env.Var = Parameter 2, estimate, confidence level, confidence low, confidence high, statistic, degrees of freedom error, Benjamin-Hochberg corrected *p*-value, *p*-value adjusted method, method used, number of observations. Significant correlations are marked in red.

**A separate Excel file has been uploaded.**

**Supplemental Table 7.** Significant differential abundant KO identifier predictions on ASVs of bottom water (BW) and sediment (SED) samples which were significant differential abundant between the heated and control bay, over 1 % relative abundant, and found in at least 50 % of the samples using the PICRUST2 and ALDEx2 tools. Shown are: KO-identifier, rab.all= media clr value, rab.win.control= median clr value for control samples, rab.win.heated= median clr value for heated bay samples, diff.btw= median differences in clr values, diff.win= median of the largest differences, effect= median effect size, overlap = proportion of effect size that overlaps 0, we.ep=*p*-value of Welch´s t test, we.eBH= Benjamin- Hochberg corrected *p*-value of Welch´s t test, wi.ep= *p*-value of Wilcoxon rank test, wi.epBH= Benjamin-Hochberg corrected *p*-value of the Wilcoxon rank test, gene info = KO identifier gene information.

**A separate Excel file has been uploaded.**


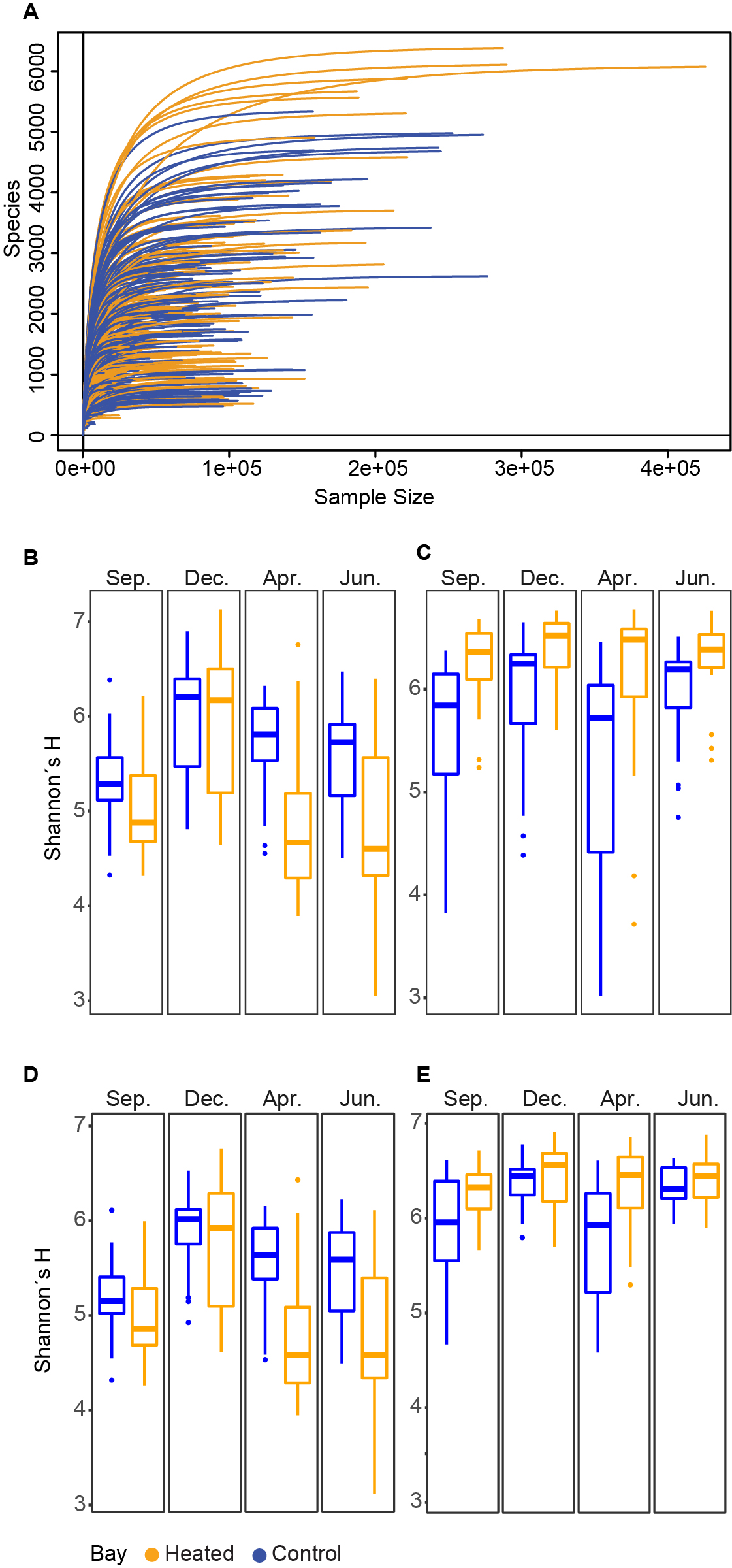


**Supplementary Figure 1.** (A) Rarefaction curve of raw counts of bottom water and sediment (*n*= 381) for the heated (orange) and control (blue) bay; Shannon´s diversity index after exclude all single and doubletons within the dataset of (B) bottom water and (C) sediment samples; Shannon´s H diversity index after rarefying the dataset to the smallest sample size (*n*=381) within the dataset of (D) bottom water and (E) samples.

**
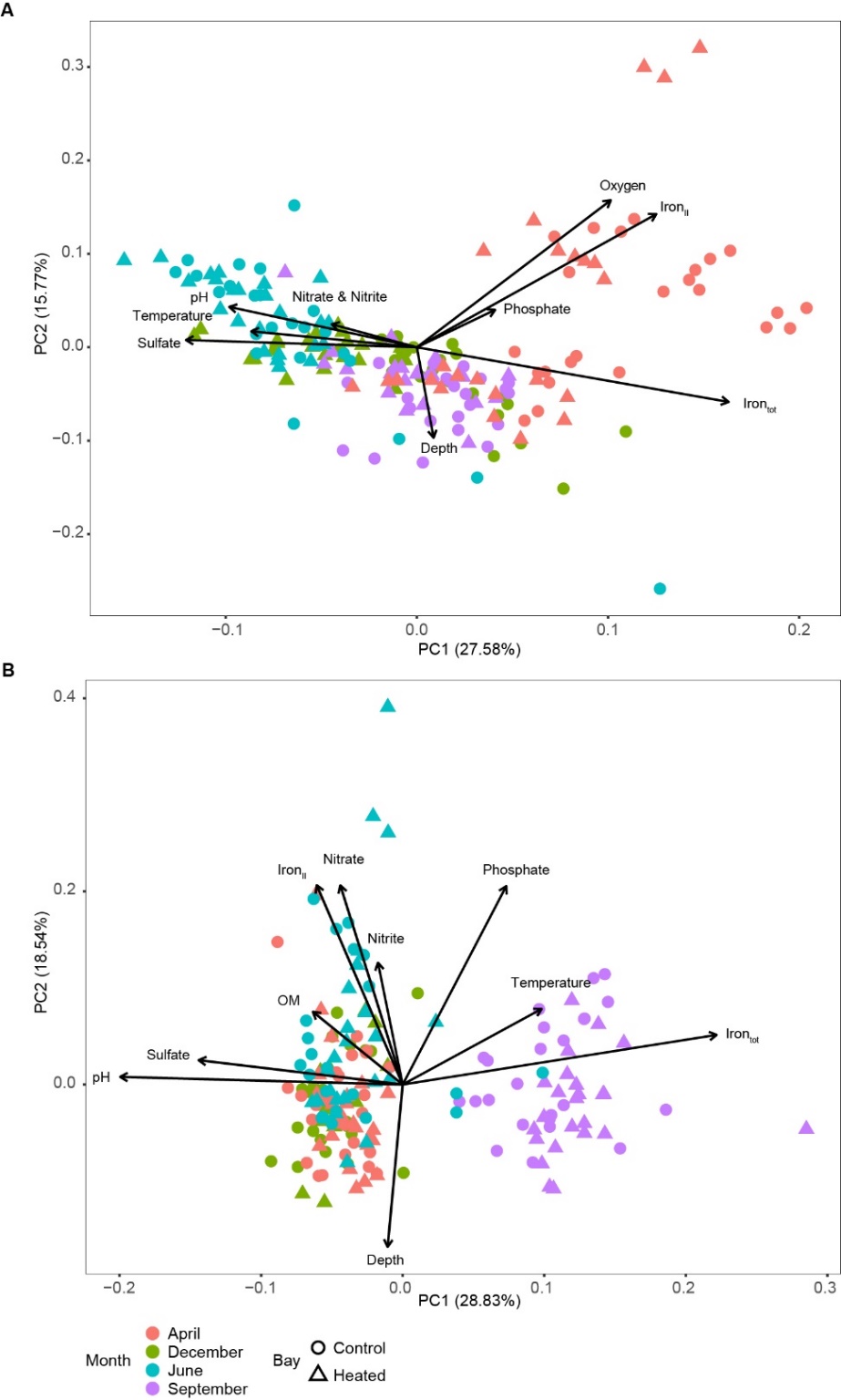
**

**Supplementary Figure 2.** (A) PCA of environmental variables measured within bottom water samples in the heated (triangle; *n*=96) and control (circle; *n*=96) bay within the month September (purple), December (green), April (red), and June (blue). Included environmental variables were: oxygen, temperature, pH, nitrate & nitrite (in combination), total iron, ferrous iron, sulfate, phosphate, and depth; (B) PCA of environmental variables measured within surface sediment samples in the heated (triangle; *n*=96) and control (circle; *n*=96) bay within the month September (purple), December (green), April (red), and June (blue). Included environmental variables were: nitrate, nitrite, sulfate, total iron, ferrous iron, pH, organic matter (OM), temperature, and depth.

**
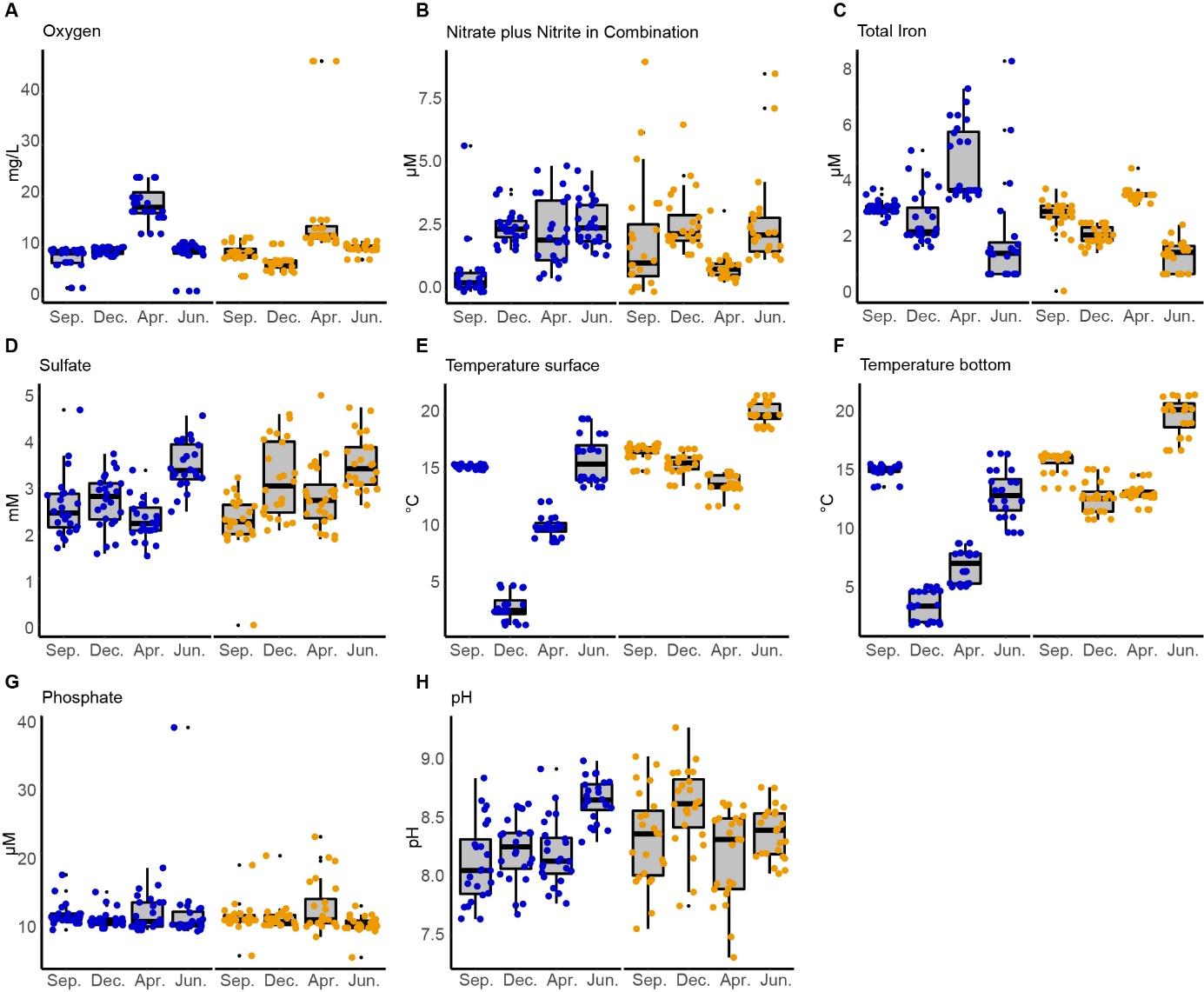
**

**Supplementary Figure 3.** Boxplots of the measured environmental variables of samples from bottom water of the heated (orange; *n*=96 for eight sites as triplicates within four sampling month and *n*=32 for oxygen and temperature at eight sites within four sampling month, respectively) and control (blue; *n*=96 and *n*=32) bay for the four different sampling month (September, December, April, and June). (A) Oxygen (*n*=60), outlier sample X13, X14, X15, X2013, X2014, X2015 were excluded, for better visualization (Supplemental Table S1); (B) Nitrate plus nitrite in combination (*n*=186); (C) Total iron (*n*=173); (D) Sulfate (*n*=191); (E) Temperature surface (*n*=64), (F) Temperature bottom (*n*=64); (G) Phosphate (*n*=191), outlier sample X2081 was excluded, for better visualization (Supplemental Table S1); (H) pH (*n*=191).

**
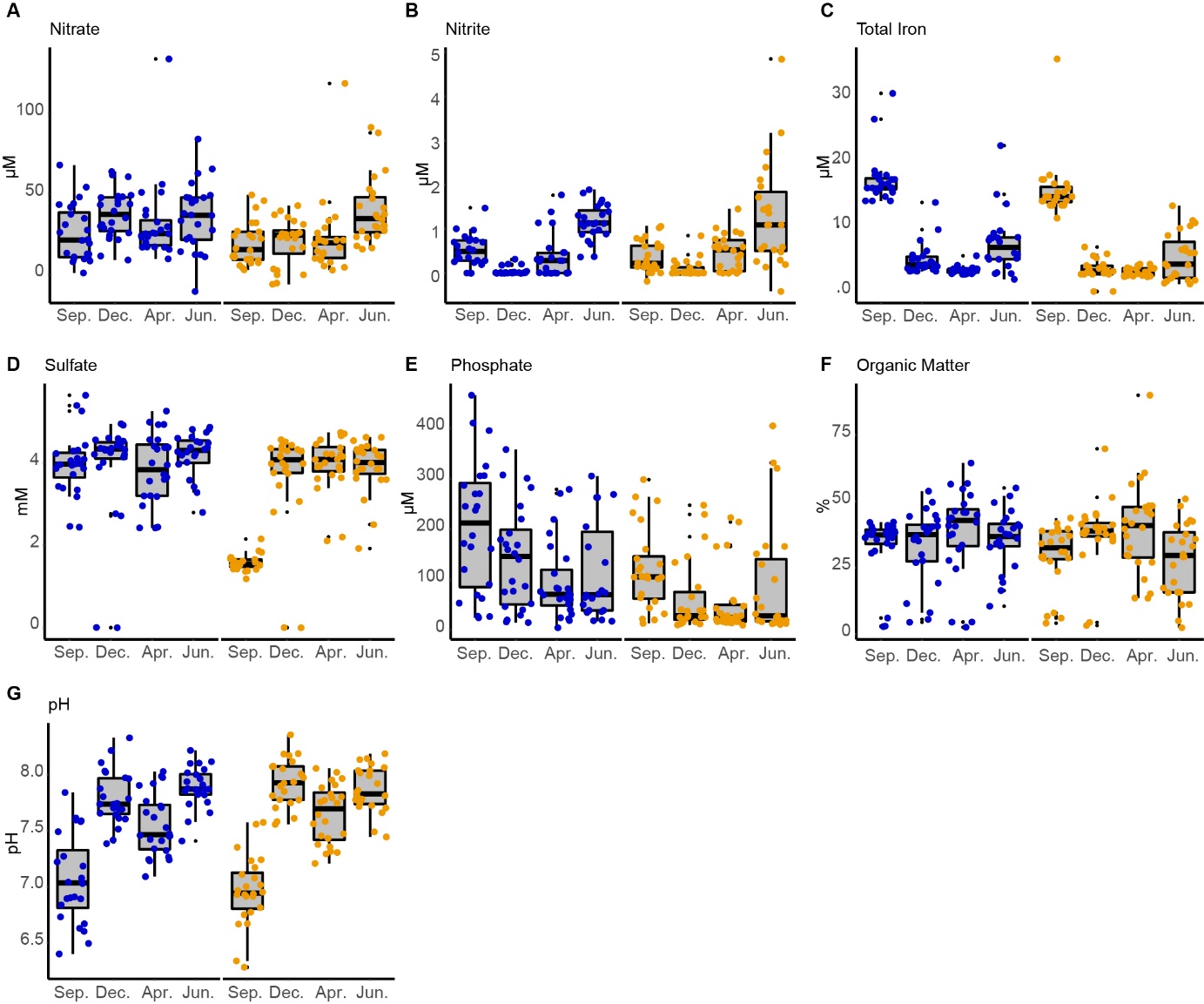
**

**Supplementary Figure 4.** Boxplots of the measured environmental variables of samples from surface sediment of the heated (orange; *n*=96) and control (blue; *n*=96) bay for the four different sampling month (September, December, April, and June). (A) Nitrate (*n*=187); (B) Nitrite (*n*=137), outlier sample X70 was excluded, for better visualization (Supplemental Table S1); (C) Total iron (*n*=184); (D) Sulfate (*n*=188); (E) Phosphate (*n*=188); (F) Organic matter (*n*=192); (G) pH (*n*=189).

**
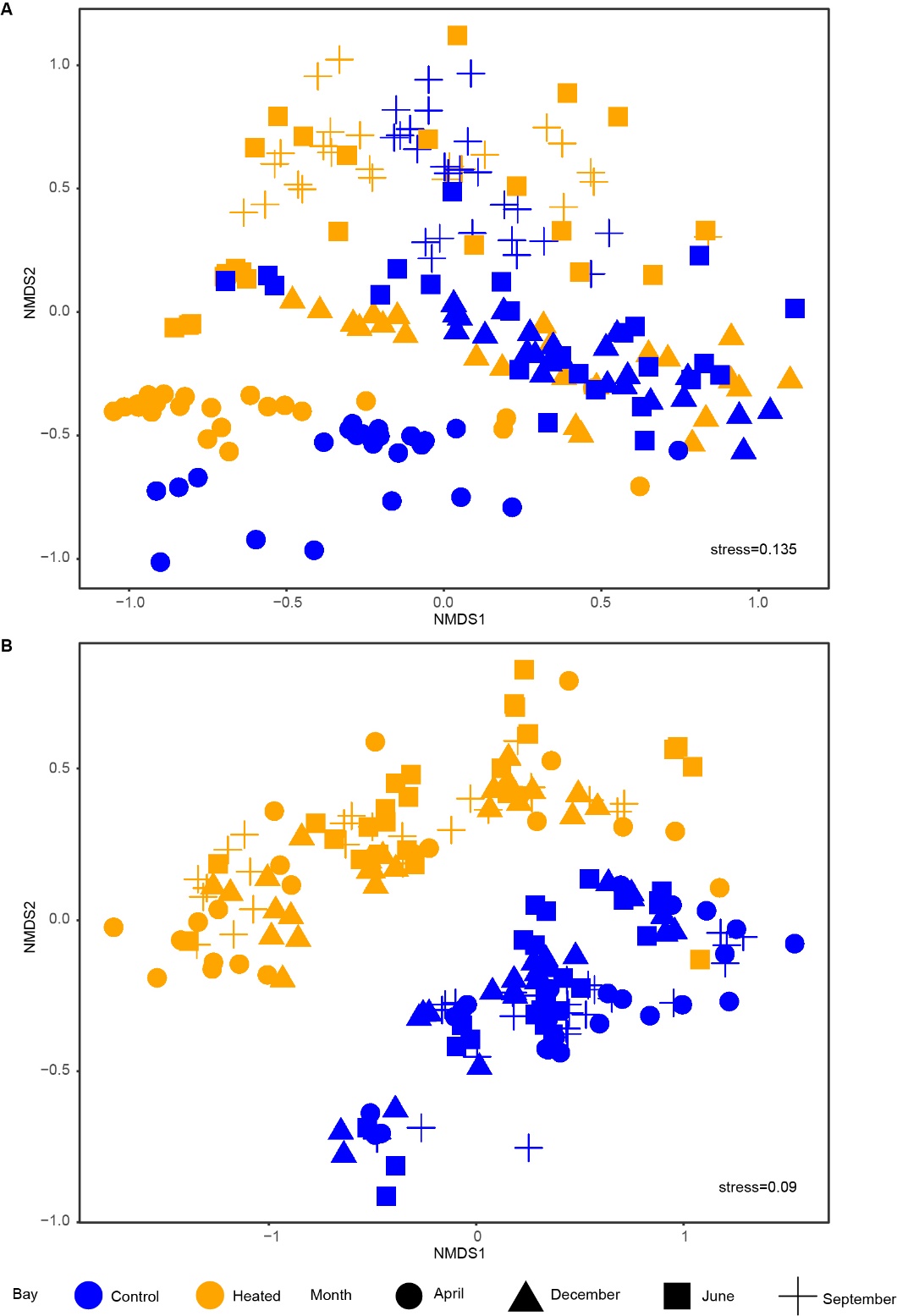
**

**Supplementary Figure 5.** Non-metric multi-dimensional scaling (nMDS) of the collected bottom water (A) and surface sediment (B) samples in the heated (orange; *n*=95 each bottom water and sediment, respectively) and control (blue; *n*=96) bay. Shown are the samples collected at the different locations within the sampling month September (cross), December (triangle), April (circle), and June (square).

**
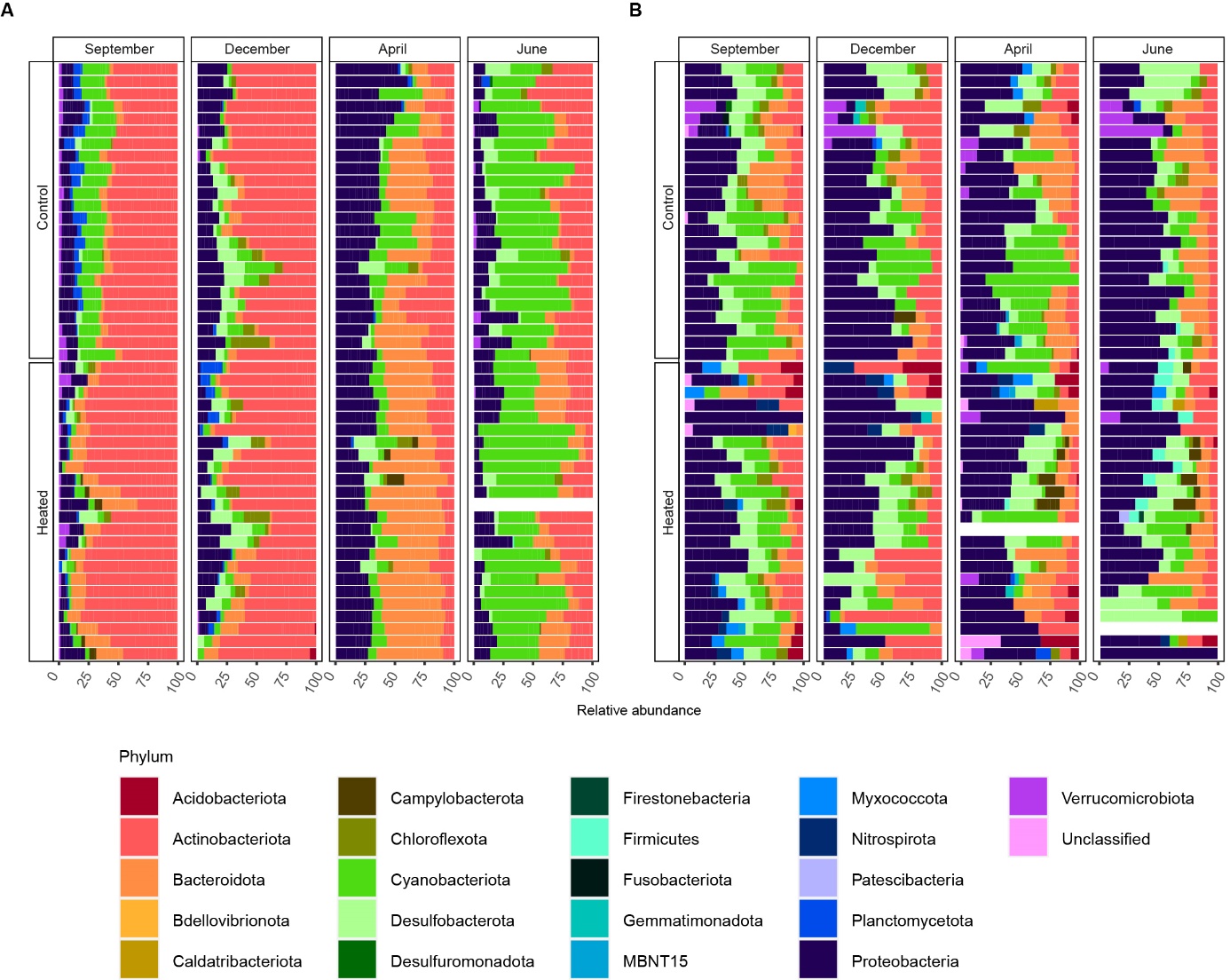
**

**Supplementary Figure 6.** Barplot of summarized ASVs above 0.5 % relative abundance on phylum level. (A) Shows the observed taxa within the bottom water samples (*n*=191) during the months September, December, April, and June in the control and heated bays, respectively. One sample within the heated bay in June was removed due to too low sequencing depth. (B) Shows the observed taxa within the sediment samples (*n*=191) during the months September, December, April, and June in the control and heated bays, respectively. One sample within the heated bay in June and April was removed due to too low sequencing depth.


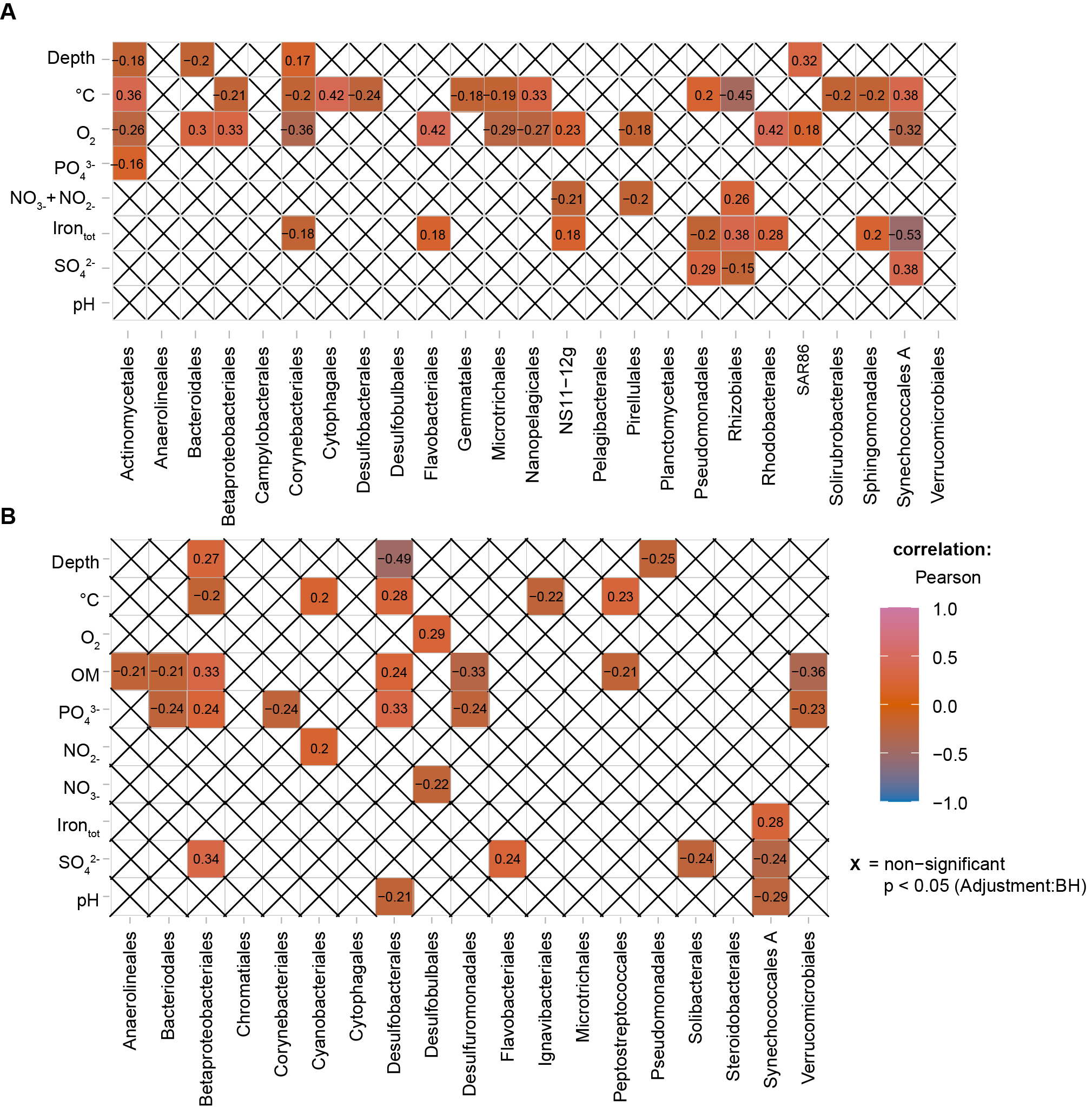


**Supplementary Figure 7.** Pearson correlations. Correlation of taxa on order level and environmental variables in bottom water (A) and sediment (B) samples. Correlation factor between 1 (pink) and -1 (blue) indicate positive and negative correlation between the parameters, respectively. Non-significant Benjamin-Hochberg *p*-value corrected correlations are indicated with an X.
